# COVID-19 preparedness—a survey among neonatal care providers in low- and middle-income countries

**DOI:** 10.1038/s41372-021-01019-4

**Published:** 2021-04-13

**Authors:** Claus Klingenberg, Sahil K. Tembulkar, Anna Lavizzari, Charles C. Roehr, Danielle E. Y. Ehret, Nestor Eduardo Vain, Gonzalo Luis Mariani, Omer Erdeve, Victor Javier Lara-Diaz, Sithembiso Velaphi, Hon Kin Cheong, Surender Singh Bisht, Khawaja Ahmad Irfan Waheed, Alexander G. Stevenson, Nisreen Al-Kafi, Jean-Michel Roue, Alejandra Barrero-Castillero, Eleanor J. Molloy, John A. F. Zupancic, Jochen Profit, The International Neonatal COVID-19 Consortium

**Affiliations:** 1 Paediatric Research Group, Department of Clinical Medicine, UiT—The Arctic University of Norway, Tromso, Norway; 2 Department of Paediatric and Adolescent Medicine, University Hospital of North Norway, Tromso, Norway; 3 Department of Pediatrics, Division of Neonatal and Developmental Medicine, Stanford University School of Medicine, Palo Alto, CA USA; 4 California Perinatal Quality Care Collaborative, Palo Alto, CA USA; 5 Fondazione IRCCS Ca’ Granda Ospedale Maggiore Policlinico, NICU, Milan, Italy; 6 Newborn Services, John Radcliffe Hospital, Oxford University Hospitals NHS Foundation Trust, Oxford, UK; 7 National Perinatal Epidemiology Unit, Nuffield Department of Population Health, Medical Sciences Division, University of Oxford, Oxford, UK; 8 Department of Pediatrics, Robert Larner MD College of Medicine, University of Vermont, Burlington, VT USA; 9 Vermont Oxford Network, Burlington, VT USA; 10 Newborn Medicine, Hospitals Sanatorio Trinidad, Buenos Aires, Argentina; 11 Department of Pediatrics, Division of Neonatology, Instituto Universitario Hospital Italiano de Buenos Aires, Buenos Aires, Argentina; 12 Department of Pediatrics, Division of Neonatology, Ankara University School of Medicine, Ankara, Turkey; 13 Tecnologico de Monterrey, Escuela de Medicina y Ciencias de la Salud, Monterrey, NL Mexico; 14 Department of Paediatrics, Faculty of Health Sciences, University of the Witwatersrand, Johannesburg, South Africa; 15 Department of Paediatrics, KPJ Ipoh, KPJ Healthcare Berhad, Kuala Lumpur, Malaysia; 16 Neonatal Intensive Care Unit, Swami Dayanand Hospital, Delhi, India; 17 The Children’s Hospital & The Institute of Child Health, Lahore, Pakistan; 18 Department of Paediatrics and Child Health, University of Zimbabwe, Harare, Zimbabwe; 19 Division of Neonatology, King Fahad Armed Forces Hospital, Jeddah, Saudi Arabia; 20 Department of Pediatrics, Division of Neonatal and Pediatric Critical Care Medicine, University Hospital of Brest, Brest, France; 21 Department of Neonatology, Beth Israel Deaconess Medical Center, Boston, MA USA; 22 Department of Pediatrics, Division of Newborn Medicine, Harvard Medical School, Boston, MA USA; 23 Paediatrics, Academic Centre, Children’s Hospital Ireland at Tallaght, Trinity College, the University of Dublin, Dublin, Ireland; 24 Trinity Translational Medicine Institute & Trinity Research in Childhood Centre, Dublin, Ireland; 25 Department of Neonatology, Coombe Women and Infants’ University Hospital, Dublin, Ireland

**Keywords:** Paediatrics, Health services

## Abstract

**Objective:**

To evaluate COVID-19 pandemic preparedness, available resources, and guidelines for neonatal care delivery among neonatal health care providers in low- and middle-income countries (LMICs) across all continents.

**Study design:**

Cross-sectional, web-based survey administered between May and June, 2020.

**Results:**

Of 189 invited participants in 69 LMICs, we received 145 (77%) responses from 58 (84%) countries. The pandemic provides significant challenges to neonatal care, particularly in low-income countries. Respondents noted exacerbations of preexisting shortages in staffing, equipment, and isolation capabilities. In Sub-Saharan Africa, 9/35 (26%) respondents noted increased mortality in non-COVID-19-infected infants. Clinical practices on cord clamping, isolation, and breastfeeding varied widely, often not in line with World Health Organization guidelines. Most respondents noted family access restrictions, and limited shared decision-making.

**Conclusions:**

Many LMICs face an exacerbation of preexisting resource challenges for neonatal care during the pandemic. Variable approaches to care delivery and deviations from guidelines provide opportunities for international collaborative improvement.

## Introduction

The COVID-19 pandemic has hit the global community with disastrous consequences for health, economic, and social structures, not seen for the last century [1, 2]. The pandemic has strained health care capacities in high-income countries (HICs) [3, 4]. Low- and middle-income countries (LMICs) face even greater challenges with overwhelmed health care systems and substantial excess deaths [5–7]. The political environment, health system capacity, and ethnic and social inequality are among the factors potentially explaining differences in outcomes within and between countries and regions [8].

Neonatal COVID-19 infections continue to be infrequent. Vertical transmission, independent of mode of birth, is rare, and postnatal infections are equally common in breastfed and formula-fed infants [9]. Despite intense research [10–12], it remains unclear why neonates mainly experience mild symptoms and have low mortality rates. At present, the pandemic’s indirect effects, with diversion of resources, shortage of qualified perinatal staff, and fear among pregnant mothers to seek health care, are arguably of greater concern for global neonatal health [13, 14].

In HICs, variation in guidelines and care practices exists for neonates born to mothers with COVID-19 [15]. Controversial issues involve maternal–infant separation, breastfeeding, the “need for” personal protective equipment (PPE) in handling of babies, and isolation routines [9, 15, 16]. Little is known regarding pandemic preparedness and care ramifications in LMICs, where the vast majority of births and 99% of global neonatal mortality occur [17]. The World Health Organization (WHO) and many LMICs have issued perinatal COVID-19 guidelines [18–20], but local response may differ substantially according to health system organization and availability of resources [7, 21]. To better understand the variation in COVID-19 pandemic preparedness, available resources, and modifications to care delivery, we conducted a survey among neonatal health care providers in LMICs. We aim to present global data from resource-limited settings, to aid hospital administrators and stakeholders with service planning and allocation of resources.

## Methods

### Survey design and translation

This study follows on our prior survey describing neonatal COVID-19 guidelines in 20 predominantly HICs in March 2020 [15]. In the current study, we targeted neonatal care providers in LMICs (low income, lower middle income, and upper middle income), according to World Bank income category classification [22]. We identified and contacted leading neonatologists from LMICs on all continents through peer networking and pediatric societies’ member lists. Nine of these agreed to participate as “regional leaders” (SV, AGS, NA-K, OE, HKC, SSB, KAIW, NEV, GLM). The initial survey draft was circulated among all co-authors and was adapted according to written and oral feedback, during webinar group discussions and e-mails over a 5-week period until consensus. We pilot tested the online survey for clarity, readability, and functionality with four neonatal professionals with different levels of experience and from different workplaces, broadly representative of the target settings. Neonatologists from Mexico and France translated the English version into Spanish and French, respectively.

### Survey content

The survey questionnaire (Appendix) requested information about the demographics and epidemiological data for each respondent’s hospital and neonatal unit. We then asked about the effect of COVID-19 on the neonatal unit, isolation capabilities, access to resources including viral testing, visitor restrictions, delivery room management, hygiene precautions in the neonatal unit, and practices on separation, feeding, and discharge for asymptomatic infants born to mothers with COVID-19. Finally, we invited respondents to list potential innovations emerging during the pandemic. Survey response options varied according to domain, and included discrete or multiple-choice options, a five-point Likert scale ranging from “disagree strongly” to “agree strongly,” and open-ended questions. Text boxes throughout the survey provided an opportunity for respondent comments.

### Survey administration

Each regional leader attempted to identify a minimum of two practicing neonatal care providers in countries within their own region as possible survey respondents. For each region, we sought to reach respondents practicing in a variety of care delivery systems, including urban, rural, public, or private settings. The web-based survey (Qualtrics XM^TM^, Provo, UT, USA) was distributed to 189 neonatal care providers in 69 LMICs between May 27 and June 17, 2020. Nonresponders received up to three reminder messages.

### Analysis and approval

We conducted descriptive analyses and histograms to evaluate survey responses. We used the chi-square test to analyze differences between countries grouped in the low-income country (LIC), lower-middle-income country (lower-MIC), and upper-middle-income country (upper-MIC) categories. Data were analyzed using Microsoft Excel and Qualtrics XM^TM^ reporting outputs. The study received approval from the Stanford Institutional Review Board (Protocol # 56237) as not meeting criteria for human subjects research.

## Results

We received 145 individual responses (response rate 77%) from 58 LMICs (84% of the invited LMICs). We have categorized and report the responses by five large global regions and/or by the three income categories (Supplementary Fig. 1). In Latin America and the Caribbean, the Middle East and North Africa, and Europe and Central Asia, most responses came from countries belonging to the upper-MICs, whereas almost half of the Sub-Saharan African countries were in the LICs (Supplementary Fig. 1). Most respondents worked in regional referral centers, mainly public hospitals, with over 3000 annual deliveries and over 500 annual admissions to their neonatal unit (Table 1).

**Table 1:** Background demographics of hospital and neonatal unit, and perceived impact of the COVID-19 pandemic.

	South-East Asia *n* = 23	Europe and Central Asia *n* = 25	Middle East and North Africa *n* = 23	Latin America and Caribbean *n* = 39	Sub-Saharan Africa *n* = 35
Public hospital^a^	16 (70%)	19 (76%)	18 (78%)	23 (59%)	28 (80%)
Neonatal unit category					
Regional referral center	16 (70%)	12 (48%)	17 (74%)	31 (79%)	28 (80%)
Other	7 (30%)	13 (52%)	8 (26%)	8 (21%)	7 (20%)
>3000 annual deliveries in hospital	11 (48%)	19 (76%)	10 (43%)	21 (54%)	20 (57%)
>500 annual admission to neonatal unit	17 (74%)	19 (76%)	14 (61%)	22 (56%)	23 (66%)
Respiratory care available					
Mechanical ventilation	21 (91%)	25 (100%)	21 (91%)	38 (97%)	13 (37%)
Noninvasive support	1 (4.5%)	0	1 (4.5%)	1 (3%)	17 (49%)
Only oxygen	1 (4.5%)	0	1 (4.5%)	0	5 (14%)
Isolation possibilities					
No single rooms	9 (39%)	5 (20%)	7 (30%)	14 (36%)	23 (66%)
Insufficient single rooms	8 (35%)	7 (28%)	9 (39%)	18 (46%)	7 (20%)
Sufficient single rooms	5 (22%)	10 (40%)	4 (17%)	7 (18%)	4 (11%)
Do not know	1 (4%)	3 (12%)	3 (13%)	0	1 (3%)
Shortage of neonatal staff^b^	7 (30%)	13 (52%)	14 (61%)	28 (72%)	19 (54%)
Admission rates^b^					
Increased	3 (13%)	4 (16%)	2 (9%)	5 (13%)	4 (11%)
Decreased	5 (22%)	9 (36%)	7 (39%)	15 (38%)	13 (37%)
No change/do not know	15 (65%)	12 (48%)	14 (61%)	19 (49%)	18 (52%)
Increased mortality for non-COVID-19-infected infants^b^					
Yes	4 (17%)	2 (8%)	1 (4%)	2 (5%)	9 (26%)
No	18 (79%)	22 (88%)	13 (57%)	34 (87%)	22 (63%)
Do not know	1 (4%)	1 (4%)	9 (39%)	3 (8%)	4 (11%)

^a^Nonpublic hospitals included private hospital for profit and not for profit and other.

^b^Perceived impact during the pandemic. Staff included nurses and/or doctors.

**Fig. 1:**
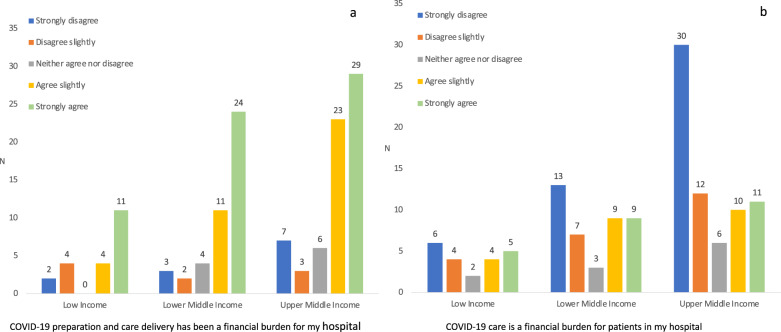
Responses to survey questions. **a** “COVID-19 preparation and care delivery has been a financial burden for my hospital,” and **b** “COVID-19 care is a financial burden for patients in my hospital.”

The majority of respondents in all regions, except in South-East Asia, reported a shortage of neonatal staff during the pandemic (Table 1), some of them specifying that this was prevalent even before the pandemic. The admission rates to neonatal units were overall not changed (74/145; 51%) or even decreased (49/145; 34%), in particular in Sub-Sahara African (Table 1). Reported reasons for decreased admission rates were reductions in perinatal care capacity, isolation or quarantining of health care workers, delayed or reduced referrals for delivery due to travel restrictions, and fear among women to seek hospital health care during the pandemic. COVID-19 was perceived as a financial burden to many hospitals, in particular in Latin America and the Caribbean and Sub-Saharan African (Fig. 1a). However, most respondents reported that increased hospital costs did not put any extra financial burden on patients in their hospital (Fig. 1b). Nine out of 35 (26%) respondents from Sub-Saharan Africa reported that they had observed an increased mortality rate for non-COVID-19-infected infants in their unit, due to shortage of equipment and staff, and reduced referrals. Increased infant mortality was less commonly reported from other regions (*p* < 0.001). Nevertheless, nine other respondents, predominantly from public hospitals (8/9), in LICs (*n* = 3), lower-MICs (*n* = 3), and upper-MICs (*n* = 3) had observed increased non-COVID mortality in neonates. Actual mortality estimates were not obtained.

**Fig. 2:**
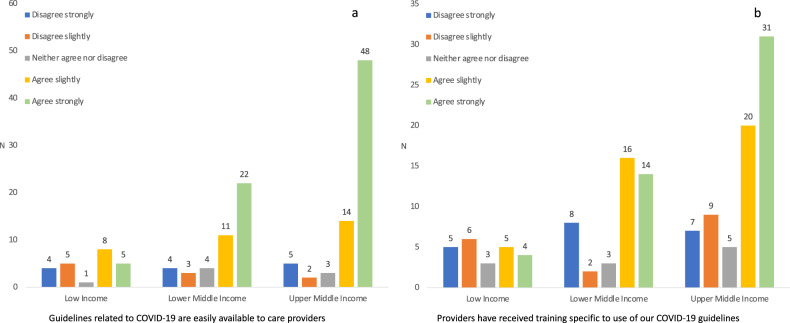
Responses to survey questions. **a** “Guidelines related to COVID-19 are easily available to care providers,” and **b** “Providers have received training specific to use of our COVID-19 guidelines.”

**Fig. 3:**
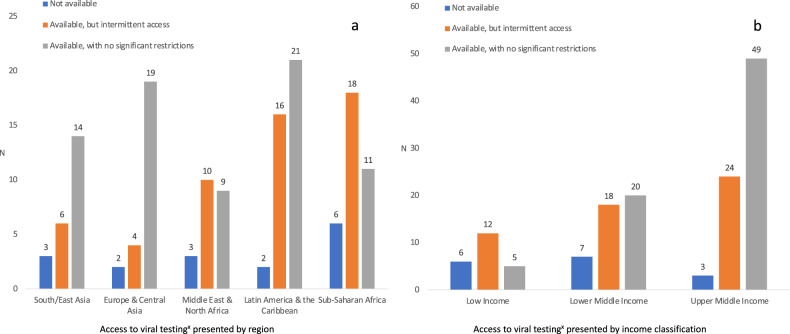
Access to viral testing. **a** Presented by region and **b** Presented by income classification. ^x^Viral testing was reported to be dependent on the family’s ability to pay for testing from total of 11/145 hospital respondents: 2 respondents in South-East Asia, 3 respondents in Europe and Central Asia, 0 respondents in Middle East and North Africa, 4 respondents in Latin-America and Caribbean, and 2 respondents in Sub-Saharan Africa.

The majority of respondents reported that their unit followed either WHO, national, or local guidelines for the care of infants born to COVID-19 positive mothers (Table 2). The guidelines were easily available in most regions except in LICs (Fig. 2a), but specific training in relation to guidelines was less common (Fig. 2b). Viral testing was available, free of charge, in many hospitals but less common in LICs (*p* < 0.001) (Fig. 3). Additional testing with either blood PCR or serology was reported by 32 (22%) and 17 (12%) respondents, respectively (data not shown).

**Table 2:** Guidelines, recommendations, and neonatal care practices during the COVID-19 pandemic.

	South-East Asia *n* = 23	Europe and Central Asia *n* = 25	Middle East and North Africa *n* = 23	Latin America and Caribbean *n* = 39	Sub-Saharan Africa *n* = 35
Guidelines followed or in use^a^					
WHO	10 (43%)	14 (56%)	16 (70%)	14 (36%)	15 (43%)
National	17 (74%)	19 (76%)	8 (35%)	19 (49%)	19 (54%)
Local	11 (48%)	7 (28%)	8 (35%)	27 (69%)	16 (46%)
Viral testing					
Not available	3 (13%)	2 (8%)	4 (17%)	2 (5%)	6 (17%)
Available, intermittent access	6 (26%)	4 (16%)	10 (43%)	16 (41%)	18 (51%)
Available, no restrictions	14 (61%)	19 (76%)	9 (40%)	21 (54%)	11 (32%)
Delivery room management					
Immediate cord clamping	11 (48%)	15 (60%)	8 (35%)	19 (49%)	7 (20%)
Delayed cord clamping	4 (17%)	7 (28%)	3 (13%)	17 (44%)	12 (34%)
Do not know/missing	8 (35%)	3 (12%)	12 (52%)	3 (7%)	16 (46%)
Separate asymptomatic infant from mother with COVID-19	6 (26%)	14 (56%)	11 (47%)	15 (38%)	7 (20%)
Feeding of asymptomatic infant born to mother with COVID-19					
Breastfeeding	14 (61%)	12 (48%)	10 (43%)	19 (49%)	21 (60%)
Expressed breast milk	5 (22%)	8 (32%)	7 (30%)	16 (41%)	9 (26%)
Formula	3 (13%)	5 (20%)	6 (26%)	3 (8%)	2 (6%)
Do not know	1 (4%)	0	0	1 (3%)	3 (9%)
Discharge of asymptomatic infant born to mother with COVID-19					
As before	11 (48%)	4 (16%)	6 (26%)	25 (64%)	15 (43%)
Later (for observation)	6 (26%)	9 (36%)	5 (22%)	3 (8%)	10 (29%)
Earlier (to avoid transmission)	2 (9%)	7 (28%)	9 (39%)	10 (20%)	5 (14%)
Do not know	4 (17%)	5 (20%)	3 (13%)	1 (3%)	5 (14%)

^a^Multiple responses possible. Total may exceed 100% for this item.

Running water, soap, and clean towels were available in most neonatal units, but three respondents from Sub-Saharan Africa and one from South-East Asia reported preexisting lack of access to running water < 50% of the time (Supplementary Fig. 2a–c). Basic PPE like face masks and gloves were also mostly available, but more than half of respondents in Sub-Saharan Africa and Middle East and North Africa reported lack of advanced face masks (e.g., N95, FFP3) during the pandemic (Supplementary Fig. 2d–g). Adequate, continuous access to sanitation and PPE was more common in upper-MICs, versus lower-MICs and LICs (*p* values < 0.03) (Supplementary Fig. 2a–g). Isolation possibilities within the neonatal units were restricted due to the lack of single rooms (Table 1). Negative-pressure rooms were available in 8/145 (5.5%) hospitals, none of which were in Sub-Saharan Africa. In Sub-Saharan Africa, 6 of 35 (17%) neonatal units described practicing co-bedding of two babies in the same cot due to restricted space. Existing capacity for mechanical ventilation was higher in upper-MICs (75/77 (97%)) than in lower-MICs (32/45 (71%)) and in LICs (11/23 (48%); *p* < 0.001).

During the pandemic, early cord clamping was more common than delayed in all regions except Sub-Saharan Africa (Table 2). Respondents recommended the use of PPE with face mask, gloves, and preferably gowns for all neonatal resuscitations, with no distinction between vaginal or cesarean delivery. After delivery, the majority of respondents in upper-MICs suggested separation of the infant from a COVID-19 positive mother, whereas recommendations in lower-MICs and LICs varied (Table 2 and Supplementary Fig. 3). We asked whether they would follow the family’s wishes to let the infant stay with a COVID-19 positive mother after birth. Overall, between 1/3 and 2/3, most frequently in upper-MICs, disagreed strongly or slightly with this approach (Supplementary Fig. 4). However, in most hospitals providers advised mothers to breastfeed (Table 2 and Supplementary Fig. 5a, b). Visitor restrictions were uniformly implemented in all regions, and only one respondent, from Sub-Saharan Africa, reported no restrictions. Fathers were in general not permitted to stay with the mother unless they had a negative test.

Potential innovations or improvements emerging during the pandemic included (1) improved parental education on hand hygiene and general infection prevention guidelines, (2) the use of telemedicine for parents’ counseling, patient follow-up, staff training, and scientific communication, and (3) establishing psychological support for the health care workers to reduce stress and anxiety.

## Discussion

In this survey, we describe the current state of the neonatology community’s response to the COVID-19 pandemic in resource-limited settings in 2020. Neonatal care in LMICs during the pandemic, particularly in LICs, was challenged by staff shortage, lack of equipment, and inadequate or lacking isolation facilities. We report striking variation in recommendations regarding timing of cord clamping, breastfeeding, and maternal–infant separation, despite clear recommendations from the WHO [18]. Notably, even for domains where most respondents did not report problems, a minority consisting predominantly of units from LICs reported significant constraints, thus highlighting the critical need to recognize local context in formulating a coordinated pandemic response.

Many respondents reported decreased admission rates to the neonatal unit during the pandemic, while also reporting current staffing shortages. Decreased admissions of children during the pandemic have also been described in other low-resource settings [23]. Diversion of physical, financial, and personnel resources away from neonates and children during the pandemic is of great concern [24, 25]. The emerging acute constraints in capacity are additive to already known limitations of neonatal care in LMIC settings, exemplified by a lack of isolation rooms and a baseline unavailability of mechanical ventilation in more than half of LIC hospitals in our survey. Some neonatal units in LICs reported deficiencies in basic sanitation equipment that is considered essential to protect human health during the pandemic [21]. Modeling studies suggest that further reduction of capacities in resource-limited settings will have substantial negative effects on maternal and neonatal health [14, 26]. Indeed, increased neonatal mortality from non-coronavirus-related conditions was described by one quarter of respondents in Sub-Saharan Africa.

The scenario of a viral pandemic has been predicted for decades [6], but the global neonatology community was not well prepared for the COVID-19 pandemic. Respondents in LMICs confirmed that they were aware of guidelines, but they also reported not receiving training in implementation of guidelines. Moreover, clinical practices varied substantially between institutions, and in many cases local standards deviated from internationally and nationally recommended approaches to care.

During delivery, mothers with active COVID-19 are contagious [27, 28]. In agreement with international guidelines, the majority of respondents recommended the use of PPE in the delivery room, especially for resuscitation in the same room as the mother, with no distinction between vaginal or caesarian deliveries. In contrast to our previous survey in HICs [15] and somewhat surprisingly, most respondents, with the exception of those in Sub-Saharan Africa, preferred early versus delayed cord clamping. Chinese guidelines recommend early cord clamping to reduce a theoretically enhanced risk of vertical transmission [29]. Most other guidelines support delayed cord clamping [15, 16, 27, 30, 31]. Early cord clamping may in fact expose infants to higher risk of morbidity than the risk of neonatal COVID-19 infections itself [32, 33].

Physical separation of a SARS-CoV-2 positive mother and her asymptomatic infant immediately after birth is controversial [16]. The adverse consequences of such a practice on breastfeeding, risk of non-coronavirus infection, and bonding [34] may far outweigh the risk of horizontal transmission [9]. In China, separation was suggested as standard of care, and the American Academy of Pediatrics recommended temporary mother–infant separation [31, 35]. In contrast, both WHO and other guidelines from LMICs and HICs recommend that babies may stay with the mother if she can care for the infant [15, 18, 20, 27, 30]. In our previous study in HICs, the striking majority of countries at the early stage of the pandemic suggested benefits for the mother–infant dyad of remaining together, with hygiene precautions [15]. By contrast, we found that mother–infant separation was common across LMICs in all regions, except in Sub-Saharan Africa. This practice diverged from WHO guidelines [18], despite the majority of our respondents stating that these were followed.

Closely related to the issue of maternal–infant separation is that of breastfeeding. WHO recommended in March 2020 that infants born to mothers with COVID-19 should be fed according to standard infant feeding guidelines, while applying necessary precautions for infection prevention control [18]. Since then, a number of international guidelines or reports have suggested that breastfeeding is safe [9, 16, 30]. Avoidance of breastfeeding puts a baby at great risk for other infections and malnutrition. Despite this, only around half of respondents across continents recommended direct breastfeeding. In particular, many respondents from MICs advocated using expressed breast milk or formula. In contrast, only one respondent from a LIC suggested using formula, which was reassuring given the strong association of formula feeding with increased infant mortality in LICs, tragically highlighted during the HIV-epidemic two decades ago [36].

Restriction of parental access was almost uniformly implemented in our sample, and less than half of the units endorsed a shared decision-making model for separation of asymptomatic infants. Parental access restriction may reduce opportunities for skin-to-skin care, with significant detrimental effects on infant survival, infant cognitive development, and parental attachment and well-being [37, 38].

Any policy changes stemming from this study regarding the global neonatology COVID-19 response must take into account certain limitations. First, the analysis is based on a convenience sample of countries, and of institutions within those countries. Second, the results were obtained at a single time point (May–June, 2020), at which countries were at different stages in the evolution of the pandemic. Thus, conclusions regarding capabilities, preferences, and resource availability that we are ascribing to regional- or income-related differences may in fact be affected by the stage of the epidemic in those subgroups. The resulting potential bias in our survey is partially offset by the large number of countries, by the inclusion of regional collaborators, by analyzing the main outcomes by countries grouped according to World Bank income category classification, and by explicitly attempting to target a range of regional institutions to ensure a representative sample. Finally, it should be emphasized that this cross-sectional, descriptive study was not intended to determine which guidelines or recommendations are the most appropriate. Indeed, the broad range of reported challenges and capabilities suggest that the ideal approach might be for a given setting to select the most applicable and feasible aspects of the available recommendations, and to concentrate on standardizing local practice accordingly.

## Conclusion

The global community of clinicians providing care to newborns in LMICs during the COVID-19 pandemic faces significant challenges related to resource availability and training in guidelines adapted to local circumstances. These difficulties are borne disproportionately by practitioners in LICs and by families subject to restrictions on time with their infants. Mitigation of such challenges requires awareness that they exist, resources, and global coordination [39] and collaboration toward the development of newborn-specific guidance and intervention.

## Supplementary information

Appendix Survey (English)

Survey (French)

Survey (Spanish)

Supplementary Figure 1

Supplementary Figure 2a

Supplementary Figure 2b

Supplementary Figure 2c

Supplementary Figure 2d

Supplementary Figure 2e

Supplementary Figure 2f

Supplementary Figure 2g

Supplementary Figure 3

Supplementary Figure 4

Supplementary Figure 5a

Supplementary Figure 5b

Supplementary figure legends

## Data Availability

The raw data supporting the conclusion of this paper will be made available by the authors, without undue reservation, to any qualified researcher.
