# Supplementary material for: COVID-19 preparedness—a survey among neonatal care providers in low- and middle-income countries
Source: J Perinatol. 2021 Apr 13;41(5):988–97. doi: 10.1038/s41372-021-01019-4 (PMC8042838; doi:10.1038/s41372-021-01019-4)
Supplement: Supplementary file 1 — Appendix Survey (English) [file 41372_2021_1019_MOESM1_ESM.pdf]

COVID-19 Neonatal Guidelines Survey - English

Start of Block: Introduction

Dear Colleague

COVID-19 is a global disaster also affecting those of us involved in perinatal/neonatal care.

Fortunately, neonates do not seem to belong to the highest risk group for severe COVID-19 infections, but due to their immature immune system we still have to be cautious when caring for this vulnerable population.

WHO and other neonatal societies have issued guidelines on how to manage neonates born to COVID- 19 positive mothers.

The aim of this survey is to identify guidelines and preparedness for COVID-19 among neonatal health care providers worldwide, including in low-resource settings where most newborn babies are born. This may contribute to an increased focus on settings where the global community needs to allocate more focus and resources. **Please respond as to the CURRENT state in your hospital. If there are any questions that are "too sensitive", please choose "Don't know" among the answer options.**

We aim to publish the results of this survey in an International Medical Journal.

All who respond to this survey will be mentioned as "International collaborators" in the upcoming publication. All data will be handled carefully not to identify any personal or other sensitive data, and data handling has been approved by the IRB at the Stanford University School of Medicine, Stanford, US.

We greatly appreciate your participation and we expect the online survey will take approximately 20 minutes to complete.

Thank you in advance!

Coordinating Group: Claus Klingenberg, Anna Lavizzari, Sithembiso Velaphi, Omer Erdeve, Hon Kin Cheong, Surender Bisht, Irfan Waheed, Alex Stevenson, Nisreen Al-Kafi, Nestor Vain, Gonzalo Mariani, John Zupancic, Charles Christoph Roehr, Danielle Ehret and Jochen Profit

Please list your name if you want to be acknowledged as an “International collaborator" (optional)

____

Please list your email address if you want to be acknowledged as an “International collaborator" (optional)

____

In what country is your hospital located?

____

In what city is your hospital located?

____

What is the name of your hospital?

____

____

____

____

____

Page Break

Please read through these definitions:

Immediately after birth the infant's SARS-CoV-2 status is unknown. We have defined the following in bullet points below:

- SARS-CoV-2 is the name of the virus that causes COVID-19
- Personal protective equipment (PPE) includes gloves, gowns, face masks, disinfectant
- Aerosol-generating procedures include nasal high-flow therapy (nHFT/HFNC), nasal continuous positive airway pressure (nCPAP), intubation and mechanical ventilation.
- Isolation means that measures are being taken to reduce contact with other infants and reduce contact with staff
- Separation from mother means that the mother and baby are separated physically (not allowed to keep the baby with her/in the same room) in order to reduce potential risk of postnatal mother-infant transmission.
- Respiratory droplet/contact isolation is defined as use of a surgical mask, gown and gloves for staff.
- Advanced respirator masks include those designated N95/KN95/FFP2/FFP3
- Neonatal unit is any unit/ward where you care for newborns requiring MORE than ROUTINE care (e.g. special care nursery, intensive care nursery, etc.)
- Maternity unit is a unit/ward in your hospital where you attend deliveries of newborns, or where mothers and newborns remain together after delivery (i.e., includes BOTH obstetric and postpartum units)

End of Block: Introduction

Start of Block: Background data

Background Data and Local epidemiology

Definitions:

Neonatal unit is any unit/ward where you care for newborns requiring MORE than ROUTINE care (e.g. special care nursery, intensive care nursery, etc.)

Annual number of births at your hospital
(Please give a range estimate)

- ○ 0
- ○ 1-1000
- ○ 1001-3000
- ○ 3001-6000
- ○ >6000

Annual number of admissions to the neonatal unit at your hospital
(Please give a range estimate)

- ○ <101
- ○ 101-500
- ○ 501-1000
- ○ 1001-2000
- ○ >2000

What is the LOWEST BIRTH WEIGHT for which you would ROUTINELY actively resuscitate

and provide neonatal care?
(Please give a range estimate)

- ○ <751
- ○ 751-1000
- ○ 1001-1500
- ○ 1501-2000
- ○ >2000

Local resources in your neonatal unit - respiratory support

- ○ We can provide invasive and non-invasive mechanical ventilation
- ○ We can provide non-invasive respiratory support (e.g. Continuous Positive Airway Pressure (CPAP), High Flow Nasal Cannula (HFNC), but not mechanical ventilation)
- ○ We can only provide extra oxygen

Page Break

Regarding your neonatal unit, approximately what proportion of patients are in the LOWEST quarter of your country's income range?

- ◯ <25%
- ◯ 25-75%
- ◯ >75%
- ◯ Don't know

Is your hospital:

- ◯ **PUBLIC** - facilities under jurisdiction or funding of national or local government or military (e.g. government hospital or health centre/post, government maternity home, other public facility)
- ◯ **PRIVATE, for-profit** - facilities that are well-defined commercial, for-profit entities (e.g. for-profit hospital, private for profit maternity home, other for-profit private facility)
- ◯ **PRIVATE, NOT for-profit** - facilities that are well-defined commercial, but NOT for-profit entities (e.g. not for-profit hospital, non-governmental organisation, foundation- or mission-hospital, not for-profit maternity home, other not for-profit private facility)
- ◯ Other (please write the type of hospital)

  ____
- ◯ Don't know

Is your hospital:

- ○ A regional referral center
- ○ A local community hospital
- ○ Other (please write the type of hospital) ____
- ○ Don't know

Page Break

COVID-19 preparation and care delivery has been a financial burden for my HOSPITAL

- Strongly disagree
- Disagree slightly
- Neither agree nor disagree
- Agree slightly
- Strongly agree
- Don't know

COVID-19 care is a financial burden for PATIENTS in my hospital

- Strongly disagree
- Disagree slightly
- Neither agree nor disagree
- Agree slightly
- Strongly agree
- Don't know

In our neonatal unit during the COVID-19 pandemic

- ☐ We have experienced a shortage of nursing staff
- ☐ We have experienced a shortage of doctors
- ☐ We have experienced a shortage of BOTH nursing staff and doctors
- ☐ We have NOT experienced a shortage of nursing staff and doctors
- ☐ Don't know
- ☐ Additional Comment: ____

Page Break

End of Block: Background data

Start of Block: General Questions related to COVID-19

General questions related to COVID-19

Definitions:
Neonatal unit is any unit/ward where you care for newborns requiring MORE than ROUTINE care (e.g. special care nursery, intensive care nursery, etc.)

At my neonatal unit we rely on the following guidelines for care of SARS-CoV-2 positive or suspected positive patients:
(check any that apply)

- ☐ WHO Guidelines
- ☐ National Guidelines
- ☐ Local/Hospital Guidelines
- ☐ ⊗None
- ☐ Additional Comment: ____

Please answer the following questions as they relate to your neonatal unit:

	Disagree strongly	Disagree slightly	Neither agree nor disagree	Agree slightly	Agree strongly	***Don't know***
Guidelines related to COVID-19 are easily available to providers (e.g., printed copy, local website, etc.)	○	○	○	○	○	○
Providers have received training specific to use of our COVID-19 guidelines	○	○	○	○	○	○
Providers mostly follow our COVID-19 guidelines	○	○	○	○	○	○

You selected "None", do you have any additional comment on guidelines for care of SARS-CoV-2 positive or suspected positive patients?
(optional)

____

____

____

____

____

Page Break

SARS-CoV-2 positive or suspected positive MOTHERS are advised to deliver at specific COVID-19 referral centers in my region/country.

- ☐ Yes, always
- ☐ Yes, when possible
- ☐ No
- ☐ Don't know
- ☐ Additional Comment ____

SARS-CoV-2 positive or suspected positive INFANTS are transferred to specific COVID-19 referral centers in my region/country.

- ☐ Yes, always
- ☐ Yes, when possible
- ☐ No
- ☐ Don't know
- ☐ Additional comment ____

Page Break

I have the impression that the COVID-19 pandemic has increased mortality for NON-COVID-19-INFECTED INFANTS in my neonatal unit.

- ○ Yes
- ○ No
- ○ Don't know

The admission rate to my neonatal unit during the COVID-19 pandemic has:

- ○ Increased
- ○ Decreased
- ○ Not changed significantly
- ○ Don't know

Reasons for increased mortality for NON-COVID-19-INFECTED INFANTS in my neonatal unit include:
(check all that apply)

- ☐ Staffing shortage
- ☐ Lack of Equipment
- ☐ Lack of personal protective equipment (PPE; gloves, gowns, masks, disinfectant)
- ☐ ⊗ Don't know
- ☐ Additional comment ____

Reasons the admission rate has decreased include:(check all that apply)

- ☐ Decreased deliveries at my hospital; maternal avoidance of COVID-19 exposure
- ☐ Decreased deliveries at my hospital; travel restrictions for mothers
- ☐ Decreased neonatal transfers; avoidance of COVID-19 exposure to neonates
- ☐ Decreased neonatal transfers; travel restrictions for families
- ☐ Decreased neonatal transfers; shortage of ambulance or staff
- ☐ Other ____

Page Break

End of Block: General Questions related to COVID-19

Start of Block: Isolation, resources, and visitor restrictions in your neonatal unit

Isolation and resources in your neonatal unit
Definitions:

Isolation means that measures are being taken to reduce contact with other infants and reduce contact with staff
Advanced respirator masks include those designated N95/KN95/FFP2/FFP3

Neonatal unit is any unit/ward where you care for newborns requiring MORE than ROUTINE care (e.g. special care nursery, intensive care nursery, etc.)

Isolation possibilities for SARS-CoV-2 positive or suspected positive infants in your neonatal unit:

- ☐ No single rooms (all rooms in neonatal unit are for multiple patients)
- ☐ Insufficient number of single rooms
- ☐ Sufficient number of single rooms
- ☐ Don't know
- ☐ Additional comment ____

Which out of the following resources are available in your neonatal unit? (check all that apply):

- [ ] Running water
- [ ] Soap
- [ ] Clean towels
- [ ] Medical gloves
- [ ] Basic face masks
- [ ] Advanced face masks (include those designated N95/KN95/FFP2/FFP3)
- [ ] Single-use gowns
- [ ] ⊗ None
- [ ] Additional comment on equipment availability

____

You selected "None". Do you have any additional comment on equipment availability? (optional)

____

____

____

____

____

Please comment on what % of time you have access to each resource in your neonatal unit:

	0%	less than 50%	more than 50%	100%
Running water	○	○	○	○
Soap	○	○	○	○
Clean towels	○	○	○	○
Medical gloves when caring for SARS-CoV-2 suspected/positive newborns.	○	○	○	○
Basic face masks when caring for SARS-CoV-2 suspected/positive newborns.	○	○	○	○
Advanced face masks when caring for SARS-CoV-2 suspected/positive newborns and possible aerosol-generating procedures.	○	○	○	○
Single-use gowns when caring for SARS-CoV-2 suspected/positive newborns.	○	○	○	○

Page Break

Visitor restrictions in your neonatal unit

Have you implemented GENERAL visitor restrictions during the current COVID-19 pandemic in your neonatal unit?

- [ ] ⊗ No additional restrictions
- [ ] ⊗ No visitation
- [ ] Restriction to number of visitors
- [ ] Only one parent allowed
- [ ] Only one parent can visit at a time
- [ ] Restrictions for non-family members
- [ ] Restriction to duration of visit
- [ ] Comment (please specify)

____

You selected "No additional restrictions". Do you have any additional comments regarding visitor restrictions during the current COVID-19 pandemic in your neonatal unit? (optional)

End of Block: Isolation, resources, and visitor restrictions in your neonatal unit

---

Start of Block: Delivery room management of baby born to SARS-CoV-2 positive/suspected mothers

Delivery room management of infants born to SARS-CoV-2 positive or suspected positive mothers

Definitions:

Maternity unit is a unit/ward in your hospital where you attend deliveries of newborns, or where mothers and newborns remain together after delivery (i.e., includes BOTH obstetric and postpartum units)

Neonatal unit is any unit/ward where you care for newborns requiring MORE than ROUTINE care (e.g. special care nursery, intensive care nursery, etc.)

My hospital has a maternity unit.

- ○ Yes
- ○ No on-site deliveries at my hospital

In your hospital or neonatal unit, what are the recommendations regarding CORD CLAMPING for infants born to SARS-CoV-2 positive or suspected positive mothers?

- ▢ Immediate
- ▢ Delayed
- ▢ Don't know
- ▢ Additional comment ____

In your hospital or neonatal unit, what are the recommendations regarding Personal Protective Equipment (PPE) for BAG-MASK VENTILATION during resuscitation of infants born to SARS CoV-2 positive or suspected positive mothers if performed OUTSIDE delivery/labor room?

- ☐ No specific protection
- ☐ Basic protection (e.g., face mask, gloves and gown) for all resuscitation
- ☐ Advanced protection (e.g., face masks, gloves, goggles/face shield and gown) for all resuscitation
- ☐ Please specify if different after vaginal delivery or Caesarean delivery ____
- ☐ Other ____

In your hospital or neonatal unit, what are the recommendations regarding Personal Protective Equipment (PPE) for BAG-MASK VENTILATION during resuscitation of infants born to SARS CoV-2 positive or suspected positive mothers if performed INSIDE delivery/labor room?

- ☐ No specific protection
- ☐ Basic protection (e.g., face mask, gloves and gown) for all resuscitation
- ☐ Advanced protection (e.g., face masks, gloves, goggles/face shield and gown) for all resuscitation
- ☐ Please specify if different after vaginal delivery or Caesarean delivery ____
- ☐ Other ____

Page Break

In your hospital or neonatal unit, what are the recommendations regarding Personal Protective Equipment (PPE) for ENDOTRACHEAL INTUBATION during resuscitation of infants born to SARS CoV-2 positive or suspected positive mothers if performed OUTSIDE delivery/labor room?

- ☐ No specific protection
- ☐ Basic protection face mask, gloves and gown for all resuscitation
- ☐ Advanced protection (e.g., face masks, gloves, goggles/face shield and gown) for all resuscitation
- ☐ Please specify if different after vaginal delivery or Caesarean delivery ____
- ☐ Other ____

In your hospital or neonatal unit, what are the recommendations regarding Personal Protective Equipment (PPE) for ENDOTRACHEAL INTUBATION during resuscitation of infants born to SARS CoV-2 positive or suspected positive mothers if performed INSIDE delivery/labor room?

- ☐ No specific protection
- ☐ Basic protection face mask, gloves and gown for all resuscitation
- ☐ Advanced protection (e.g., face masks, gloves, goggles/face shield and gown) for all resuscitation
- ☐ Please specify if different after vaginal delivery or Caesarean delivery ____
- ☐ Other ____

End of Block: Delivery room management of baby born to SARS-CoV-2 positive/suspected mothers

Start of Block: Admission to neonatal unit of baby born to SARS-CoV-2 positive/suspected mothers

Admission to neonatal unit of infants born to SARS-CoV-2 positive or suspected positive mothers

Definitions:

Aerosol-generating procedures include nasal high-flow therapy (nHFT/HFNC), nasal continuous positive airway pressure (nCPAP), intubation and mechanical ventilation.

Respiratory droplet/contact isolation is defined as use of a surgical mask, gown and gloves.

Neonatal unit is any unit/ward where you care for newborns requiring MORE than ROUTINE care (e.g. special care nursery, intensive care nursery, etc.)

- [ ] Multiple patient room
- [ ] Single patient room
- [ ] Negative pressure room
- [ ] Don't know
- [ ] Incubator/isolette
- [ ] Crib/warmer
- [ ] Don't know
- [ ] 1 baby per bed/cot
- [ ] >1 baby per bed/cot
- [ ] Don't know
- [ ] Standard hygiene (clean hands and clothes)
- [ ] Respiratory droplet/contact isolation with basic face mask
- [ ] Respiratory droplet/contact isolation with advanced respirator masks
- [ ] Don't know

What are the routine hygiene precautions in your neonatal unit when an infant DOES RECEIVE aerosol-generating procedures?

- [ ] Multiple patient room
- [ ] Single patient room
- [ ] Negative pressure room
- [ ] Don't know
- [ ] Incubator/isolette
- [ ] Crib/warmer
- [ ] Don't know
- [ ] 1 baby per bed/cot
- [ ] >1 baby per bed/cot
- [ ] Don't know
- [ ] Standard hygiene (clean hands and clothes)
- [ ] Respiratory droplet/contact isolation with basic face mask
- [ ] Respiratory droplet/contact isolation with advanced respirator masks
- [ ] Don't know

End of Block: Admission to neonatal unit of baby born to SARS-CoV-2 positive/suspected mothers

---

Start of Block: Asymptomatic infants born to SARS-CoV-2 positive or suspected positive mothers

Asymptomatic infants born to SARS-CoV-2 positive or suspected positive mothers - separation, feeding, and discharge

Definitions:

Aerosol-generating procedures include nasal high-flow therapy (nHFT/HFNC), nasal continuous positive airway pressure (nCPAP), intubation and mechanical ventilation.

Separation from mother means that the mother and baby are separated physically (not allowed to keep the baby with her/in the same room) in order to reduce potential risk of postnatal mother-infant transmission.

Neonatal unit is any unit/ward where you care for newborns requiring MORE than ROUTINE care (e.g. special care nursery, intensive care nursery, etc.)

Maternity unit is a unit/ward in your hospital where you attend deliveries of newborns, or where mothers and newborns remain after delivery (i.e., includes BOTH obstetric and postpartum units)

WHERE do you care for ASYMPTOMATIC infants born to SARS-CoV-2 positive or suspected positive mothers INITIALLY AFTER BIRTH?

- ☐ Stay with mother as routinely done previously (no single room available)
- ☐ Stay with mother in a single room, as routinely done previously
- ☐ Stay with mother in a single room, but in a crib/isolette/incubator separated by a certain distance from mother (with or without physical barrier such as curtain)
- ☐ Stay in a separate room from mother in the hospital
- ☐ Other ____
- ☐ Don't know

Can father, partner or other family member remain with ASYMPTOMATIC infants in the location you indicated in the previous question?

- ☐ Yes, without restrictions
- ☐ Yes, with restrictions on time
- ☐ No
- ☐ Don't know
- ☐ Only if father/partner/family member tested negative
- ☐ Other comment ____

Page Break

For ASYMPTOMATIC infants and SARS-CoV-2 positive or suspected positive mothers: In our maternity/neonatal unit, we follow mother’s/family’s wishes regarding whether their infant will stay with mother after birth.

- ☐ Disagree strongly
- ☐ Disagree slightly
- ☐ Neither agree nor disagree
- ☐ Agree slightly
- ☐ Agree strongly
- ☐ Don't know

Feeding of ASYMPTOMATIC infants born to SARS-CoV-2 positive or suspected positive mothers

- ☐ Recommend breastfeeding
- ☐ Do not recommend breastfeeding, but mother advised to express milk
- ☐ Recommend formula
- ☐ Don't know

For ASYMPTOMATIC infants and SARS-CoV-2 positive or suspected positive mothers: In our maternity/neonatal unit, we follow mother's/family's wishes regarding breastfeeding.

- [ ] Disagree strongly
- [ ] Disagree slightly
- [ ] Neither agree nor disagree
- [ ] Agree slightly
- [ ] Agree strongly
- [ ] Don't know

For those who recommend breastfeeding in SARS-CoV-2 positive or suspected positive mothers, what is your HYGIENE ADVICE to mothers? (Check all that apply)

- [ ] Wash hands with soap and water before feeding
- [ ] Washing breast before feeding
- [ ] Use of face covering, including mask or scarf, during breast feeding

---

WHEN do you discharge ASYMPTOMATIC infants? (assuming mothers are SARS-CoV-2 positive or suspected positive, but in a clinical condition stable for discharge)

- ☐ Timing for discharge no different from prior routine
- ☐ Recommend to stay longer in hospital for observation
- ☐ Recommend to discharge earlier to avoid possible transmission to other patients/staff
- ☐ Don't know
- ☐ Additional comment: ____

End of Block: Asymptomatic infants born to SARS-CoV-2 positive or suspected positive mothers

---

Start of Block: Viral testing and laboratory analyses

Viral testing and laboratory analyses

Is viral testing with nasopharyngeal swabs available?

- ○ Not available
- ○ Available, but intermittent access
- ○ Available, with no significant restrictions
- ○ Don't know

How quickly are viral test results typically available?

- ☐ < 6 hours
- ☐ 6-24 hours
- ☐ 24-72 hours
- ☐ > 72 hours
- ☐ Don't know
- ☐ Other ____

Is viral testing dependent on the family's ability to pay for testing?

- ○ Yes ____
- ○ No ____
- ○ Don't know

Page Break

In addition to nasopharyngeal swabs, select any additional testing that you routinely perform in infants born to SARS-CoV-2 positive or suspected positive mothers?

- ☐ Blood viral polymerase chain reaction (PCR)
- ☐ Blood serology antibodies
- ☐ Stool
- ☐ Cerebrospinal fluid
- ☐ Placenta
- ☐ Amniotic fluid
- ☐ ⊗None

None was selected, is there any additional comment on testing?
(optional)

____

____

____

____

____

End of Block: Viral testing and laboratory analyses

Start of Block: Promising ideas (i.e., with potential improvement or benefit) in neonatal unit

Promising ideas (i.e., with potential improvement or benefit) for clinical care, support, and families

Please describe briefly if you have implemented any new promising ideas for CLINICAL CARE during the COVID-19 pandemic (optional).

Please describe briefly if you have implemented any new promising ideas to SUPPORT LOCAL NEONATAL HEALTH CARE PROVIDERS during the COVID-19 pandemic (optional).

Please describe briefly if you have implemented any new promising ideas for FAMILY/PARENTAL SUPPORT during the COVID-19 pandemic (optional).
