# Supplementary material for: COVID-19 preparedness—a survey among neonatal care providers in low- and middle-income countries
Source: J Perinatol. 2021 Apr 13;41(5):988–97. doi: 10.1038/s41372-021-01019-4 (PMC8042838; doi:10.1038/s41372-021-01019-4)
Supplement: Supplementary file 2 — Survey (French) [file 41372_2021_1019_MOESM2_ESM.pdf]

COVID-19 Neonatal Guidelines Survey - French

Cher Collègue

le COVID-19 est une catastrophe mondiale qui affecte aussi ceux d'entre nous impliqués dans les soins périnatals / néonatals.

Heureusement, les nouveau-nés ne semblent pas appartenir au groupe le plus à risque pour les infections graves à COVID-19, mais en raison de leur système immunitaire immature, nous devons rester prudents lorsque nous prenons soin de cette population vulnérable.

L'OMS et d'autres sociétés savantes néonatales ont publié des recommandations concernant la prise en charge des nouveau-nés nés de mères séropositives pour le COVID-19.

Le but de cette enquête est d'identifier les recommandations et l'état de préparation des soignants et des services de santé pour la gestion du COVID-19 dans le monde entier, y compris dans les milieux à faibles ressources où la plupart des naissances ont lieu. Cela pourrait contribuer à porter une attention accrue sur les systèmes où la communauté mondiale doit attribuer plus d’attention et de ressources. **Veuillez répondre par rapport à l'état ACTUEL dans votre hôpital. S'il y a des questions jugées "trop sensibles", veuillez choisir "Ne sais pas".**

Notre objectif est de publier les résultats de cette enquête dans une revue médicale internationale.

Tous ceux qui répondront à cette enquête seront mentionnés comme « collaborateurs internationaux » dans la prochaine publication. Une attention particulière sera portée à la gestion des données afin de ne pas permettre d’identifier des données personnelles ou autres données sensibles. Le traitement des données a par ailleurs été approuvé par le Comité d’éthique à la recherche (IRB) de Stanford University School of Medicine, Stanford, États-Unis.

Nous vous remercions sincèrement pour votre participation. Nous estimons que le temps requis pour répondre au questionnaire est de 20 minutes.

Merci d'avance!

Groupe de coordination: Claus Klingenberg, Anna Lavizzari, Sithembiso Velaphi, Omer Erdeve, Hon Kin Cheong, Surender Bisht, Irfan Waheed, Alex Stevenson, Nisreen Al-Kafi, Nestor Vain, Gonzalo Mariani, Victor Javier Lara Diaz, John Zupancic, Charles Christoph Roehr, Danielle Ehret et Jochen Profit

Veuillez indiquer votre nom si vous souhaitez être cité comme «collaborateur international» (facultatif)

____

Veuillez indiquer votre adresse e-mail si vous souhaitez être cité comme « collaborateur international » (facultatif)

____

Dans quel pays est situé votre hôpital?

____

Dans quelle ville se trouve votre hôpital?

____

Quel est le nom de votre hôpital?

____

____

____

____

____

Page Break

Veuillez lire ces définitions: Immédiatement après la naissance, le statut SARS-CoV-2 du nourrisson est inconnu. Nous précisons quelques définitions ci-dessous: SARS-CoV-2 est le nom du virus qui cause la COVID-19 L'équipement de protection individuelle (Personal Protective Equipment, PPE) comprend des gants, des blouses, des masques faciaux, un désinfectant. Les procédures génératrices d'aérosols comprennent le traitement par canules nasales haut débit (nHFT / HFNC), la pression positive continue nasale ou PPC nasale (nasal Continuous Positive Airway Pressure, nCPAP), l'intubation et la ventilation mécanique. Isolement réfère aux mesures prises pour réduire le contact auprès des autres patients et les professionnels de la santé Séparation de la mère et de l'enfant réfère à la séparation physique de la mère et du nouveau-né (interdit d'être dans la même chambre) afin de réduire le risqué potentiel de transmission maternelle au nouveau-né L'isolement respiratoire de type gouttelettes / contact est défini comme l'utilisation d'un masque chirurgical, d'une blouse et de gants. Les masques respiratoires avancés comprennent ceux désignés par N95 / FFP2 / FFP3. L'unité néonatale est une unité /salle/service où vous prenez soin des nouveau-nés nécessitant PLUS que les SOINS DE ROUTINE (ex. unité de néonatologie soins courants, soins intensifs, réanimation néonatale). L'unité de maternité est une unité / salle de votre hôpital où vous assistez à l'accouchement de nouveau-nés, ou le lieu où à la fois les mères et les nouveau-nés restent après l'accouchement (c.-à-d., à la fois les salles de naissance et les unités de suites de couches, maternité, post partum).

End of Block: Introduction

Start of Block: Background data

Données de base, épidémiologie locale Définitions: L'unité néonatale est une unité /salle/service où vous prenez soin des nouveau-nés nécessitant PLUS que les SOINS DE ROUTINE (ex. unité de néonatologie soins courants, soins intensifs, réanimation néonatale).

Nombre annuel de naissances dans votre hôpital
(estimation)

- ○ 0
- ○ 1-1000
- ○ 1001-3000
- ○ 3001-6000
- ○ > 6000

Nombre annuel d'admissions dans l'unité néonatale de votre hôpital
(estimation)

- ○ <101
- ○ 101-500
- ○ 501-1000
- ○ 1001-2000
- ○ >2000

Quel est le POIDS DE NAISSANCE LE PLUS FAIBLE (grammes) pris en charge de manière et

prodigueriez des soins néonatals COURANTE?
(estimation)

- <751
- 751-1000
- 1001-1500
- 1501-2000
- >2000

Ressources locales dans votre unité néonatale - assistance respiratoire

- Nous pouvons fournir une ventilation mécanique invasive et non invasive
- Nous pouvons fournir un soutien respiratoire non invasif (par exemple, pression positive continue (CPAP), canules nasales à haut débit (HFNC)), mais pas de ventilation mécanique
- Nous ne pouvons fournir que de l'oxygène d’appoint

Page Break

Quelle est la proportion approximative de vos patients dont les revenus sont dans le quart INFÉRIEUR de la fourchette des revenus de votre pays?

- ◯ <25%
- ◯ 25-75%
- ◯ > 75%
- ◯ Ne sais pas

Votre hôpital est-il:

- ◯ **PUBLIC** - établissements relevant de la juridiction ou du financement d'un gouvernement ou d'un gouvernement national ou local (par exemple, hôpital, centre de santé ou maternité sous la tutelle du gouvernement, autre établissement public)
- ◯ **PRIVÉ, à but lucratif** - établissements clairement identifiés à but lucratif (p. Ex. Hôpital ou clinique à but lucratif, maternité privée à but lucratif, autre établissement privé à but lucratif)
- ◯ **PRIVÉ, À BUT NON LUCRATIF** - installations qui sont des entités privées définies comme telles, mais à but NON lucratif (par exemple hôpital ou clinique à but non lucratif, organisation non gouvernementale, fondation ou hôpital de mission, maternité à but non lucratif, autre établissement privé sans but lucratif)
- ◯ Autre ____
- ◯ Ne sais pas

Votre hôpital est-il:

- ○ Un centre régional de référence
- ○ Un hôpital communautaire local
- ○ Autre ____
- ○ Ne sais pas

Page Break

La préparation et la prestation de soins liée à la COVID-19 ont été un fardeau financier pour mon HÔPITAL

- Pas du tout d'accord
- Pas d’accord
- Ni d'accord ni en désaccord
- D'accord
- Tout à fait d'accord
- Ne sais pas

Les soins liés à la COVID-19 représentent un fardeau financier pour les PATIENTS de mon hôpital

- Pas du tout d'accord
- Pas d’accord
- Ni d'accord ni en désaccord
- D'accord
- Tout à fait d'accord
- Ne sais pas

Dans notre unité néonatale pendant la pandémie de COVID-19

- ☐ Nous avons vécu une pénurie d’infirmières
- ☐ Nous avons vécu une pénurie de médecins
- ☐ Nous avons vécu une pénurie d’infirmières et médecins
- ☐ Nous n’avons PAS vécu de pénurie d’infirmières ET médecins
- ☐ Ne sais pas
- ☐ Commentaire additionnel: ________________________________

Page Break

End of Block: Background data

Start of Block: General Questions related to COVID-19

Questions générales liées à la COVID-19

Définitions:

L'unité néonatale est une unité /salle/service où vous prenez soin des nouveau-nés nécessitant PLUS que les SOINS DE ROUTINE (ex. unité de néonatologie soins courants, soins intensifs, réanimation néonatale).

Dans mon unité néonatale, nous nous appuyons sur les recommandations suivantes pour les soins aux patients positifs ou suspects de SARS-CoV-2: (plusieurs choix possibles)

- ☐ Recommandations de l'OMS
- ☐ Recommandations nationales
- ☐ Recommandations locales / hospitalières
- ☒ Aucunes
- ☐ Commentaire additionnel: ____

Veuillez répondre aux questions suivantes en ce qui concerne votre unité néonatale:

	Pas du tout d’accord	Pas d'accord	Ni d'accord ni en désaccord	D'accord	Tout à fait d'accord	***Ne sais pas***
Les recommandations relatives à la COVID-19 sont facilement disponibles pour les soignants/ professionnels de santé (par exemple, copie imprimée, site Web local, intranet, etc.)	○	○	○	○	○	○
Les soignants/professionnels de santé ont reçu une formation spécifique à l'utilisation de nos recommandations COVID-19	○	○	○	○	○	○
Les fournisseurs suivent principalement nos recommandations COVID-19	○	○	○	○	○	○

Vous avez sélectionné « Aucun. » Avez-vous des commentaires additionnels sur les guides de pratique pour les patients positifs ou avec suspicion de SARS-CoV-2?(optionnel)

____

____

____

____

____

Page Break

Il est conseillé aux MÈRES positives ou suspectes de SARS-CoV-2 d'accoucher dans des centres de référence COVID-19 spécifiques dans ma région / mon pays.

- ☐ Oui, toujours
- ☐ Oui, si possible
- ☐ Non
- ☐ Ne sais pas
- ☐ Autre (veuillez préciser) ____

Les NOUVEAU-NÉS positifs ou suspects de SARS-CoV-2 sont transférés vers des centres de référence COVID-19 spécifiques dans ma région / mon pays.

- ☐ Oui, toujours
- ☐ Oui, si possible
- ☐ Non
- ☐ Ne sais pas
- ☐ Autre (veuillez préciser) ____

Page Break

J'ai l'impression que la pandémie de COVID-19 a augmenté la mortalité des autres NOUVEAU-NÉS NON INFECTÉS PAR LA COVID-19 dans mon unité néonatale.

- ○ Oui
- ○ Non
- ○ Ne sais pas

Le taux d'admission dans mon unité néonatale pendant la pandémie de COVID-19 a:

- ○ Augmenté
- ○ Diminué
- ○ Pas changé de manière significative
- ○ Ne sais pas

Les raisons de cette augmentation de la mortalité comprennent des autres NOUVEAU-NÉS NON INFECTÉS PAR LA COVID-19 dans mon unité néonatale:
(Cochez toutes les cases)

- ☐ Pénurie de personnel
- ☐ Manque d'équipement
- ☐ Manque d'équipement de protection individuelle (EPI; gants, blouses, masques, désinfectant)
- ☐ ⊗ Ne sais pas
- ☐ Autre (veuillez préciser)

____

Les raisons de cette baisse du taux d'admission comprennent (Cochez toutes les cases):

- ☐ Diminution des accouchements dans mon hôpital; évitement maternel de l'exposition au COVID-19
- ☐ Diminution des accouchements dans mon hôpital; restrictions de voyage pour les mères
- ☐ Diminution des transferts néonatals; éviter l'exposition de COVID-19 aux nouveau-nés
- ☐ Diminution des transferts néonatals; restrictions de voyage pour les familles
- ☐ Diminution des transferts néonatals; pénurie d'ambulance ou de personnel
- ☐ Autre (veuillez préciser) ____

Page Break

End of Block: General Questions related to COVID-19

Start of Block: Isolation, resources, and visitor restrictions in your neonatal unit

Isolement et ressources dans votre unité néonatale

Définitions:

Isolement réfère aux mesures prises pour réduire le contact auprès des autres patients et les professionnels de la santé Les masques respiratoires avancés comprennent ceux désignés par N95 / FFP2 / FFP3. L'unité néonatale est une unité /salle/service où vous prenez soin des nouveau-nés nécessitant PLUS que les SOINS DE ROUTINE (ex. unité de néonatologie soins courants, soins intensifs, réanimation néonatale).

Possibilités d'isolement dans votre unité néonatale:

- ▢ Pas de chambres individuelles supplémentaires (toutes les chambres de l'unité néonatale sont pour plusieurs patients)
- ▢ Nombre limité de chambres individuelles
- ▢ Assez de chambres individuelles
- ▢ Ne sais pas
- ▢ Autre (veuillez préciser) ____

Parmi les ressources suivantes, lesquelles sont disponibles dans votre unité néonatale? (Cochez toutes les cases):

- [ ] Eau courante
- [ ] Savon
- [ ] Serviettes propres
- [ ] Gants médicaux
- [ ] Masques standards
- [ ] Masques faciaux avancés (notamment ceux désignés N95 / KN95 / FFP2 / FFP3)
- [ ] Blouses à usage unique
- [ ] ⊗ Aucun
- [ ] Commentaire additionnelsur la disponibilité de l'équipement

____

Vous avez sélectionné « Aucun. » Avez-vous des commentaires additionnels sur la disponibilité de l'équipement?
(facultatif)

____

____

____

____

____

Veuillez indiquer le pourcentage de temps pendant lequel vous avez accès à chaque ressource de votre unité néonatale:

	0%	moins de 50%	plus que 50%	100%
Eau courante	○	○	○	○
Savon	○	○	○	○
Serviettes propres	○	○	○	○
Gants médicaux pour les soins aux nouveau-nés suspects / positifs du SARS-CoV-2.	○	○	○	○
Masques de base pour les soins aux nouveau-nés suspects / positifs du SARS-CoV-2.	○	○	○	○
Masques avancés pour les soins aux nouveau-nés suspects / positifs au SARS-CoV-2 et procédures susceptibles de générer des aérosols.	○	○	○	○
Blouses à usage unique lors de la prise en charge de nouveau-nés suspects / positifs du SARS-CoV-2.	○	○	○	○

Page Break

Restrictions de visite **dans votre unité néonatale**

Avez-vous implanté des restrictions GÉNÉRALISÉES sur les visites dans votre unité néonatale pendant la pandémie de COVID-19?

- ☐ ⊗ Aucune restriction additionnelle
- ☐ ⊗ Aucun visiteur n'est permis
- ☐ Restriction du nombre de visiteurs
- ☐ Seulement un parent est permis
- ☐ Un seul parent est permis à la fois
- ☐ Restrictions pour les non-membres de la famille
- ☐ Restrictions de la durée des visites
- ☐ Autre (veuillez préciser)

____

Vous avez sélectionné « aucune restriction additionnelle. » Avez-vous d'autres commentaires face aux restrictions pour les visiteurs pendant la pandémie de COVID-19 dans votre unité néonatale?
(facultatif)

____

____

____

____

Page Break

End of Block: Isolation, resources, and visitor restrictions in your neonatal unit

Start of Block: Delivery room management of baby born to SARS-CoV-2 positive/suspected mothers

Gestion de la salle d'accouchement des nourrissons nés de mères séropositives au SRAS-CoV-2 ou soupçonnées d'être positives Définitions: L'unité de maternité est une unité / salle de votre hôpital où vous assistez à l'accouchement de nouveau-nés, ou le lieu où les mères et les nouveau-nés restent après l'accouchement (c.-à-d., à la fois les salles de naissance et les unités de suites de couches, maternité, post partum).
L'unité néonatale est une unité /salle/service où vous prenez soin des nouveau-nés nécessitant plus que les soins de routine (ex. unité de néonatologie soins courants, soins intensifs, réanimation néonatale).

Mon hôpital a une maternité / unité.

- ○ Oui
- ○ Aucun accouchement sur site dans mon hôpital

Dans votre hôpital ou unité néonatale, quelles sont les recommandations concernant le CLAMPAGE DU CORDON chez les nourrissons nés de mères positives ou suspectes de SARS-CoV-2?

- ☐ Immédiat
- ☐ Différé
- ☐ Ne sais pas
- ☐ Autre (veuillez préciser) ____

Dans votre hôpital ou unité néonatale, quelles sont les recommandations relatives à l' équipement de protection individuelle (EPI) pour ASSURER UNE VENTILATION AU MASQUE pendant la réanimation si elle est effectuée À L'EXTÉRIEUR de la salle d'accouchement / de travail?

- ☐ Pas de protection spécifique
- ☐ Protection de base (p. Ex. Masque facial, gants et blouse) pour toute réanimation
- ☐ Protection avancée (par exemple, masques faciaux, gants, lunettes / visière faciale et blouse) pour toute réanimation
- ☐ Veuillez préciser s’il y a une différence entre l'accouchement par voie basse et l'accouchement par césarienne ____
- ☐ Autre ____

Dans votre hôpital ou unité néonatale, quelles sont les recommandations relatives à l' équipement de protection individuelle (EPI) pour ASSURER UNE VENTILATION AU MASQUE pendant la réanimation si elle est effectuée À L'INTÉRIEUR de la salle d'accouchement / de travail (sur une table de réanimation séparée)?

- ☐ Aucune protection spécifique si la réanimation est effectuée EN DEHORS de la salle d'accouchement / de travail
- ☐ Protection de base (p. Ex. Masque facial, gants et blouse) pour toute réanimation
- ☐ Protection avancée (par exemple, masques faciaux, gants, lunettes / visière faciale et blouse) pour toute réanimation
- ☐ Veuillez préciser s’il y a une différence entre l'accouchement par voie basse et l'accouchement par césarienne ____
- ☐ Autre ____

Page Break

Dans votre hôpital ou votre unité néonatale, quelles sont les recommandations concernant les équipements de protection individuelle (EPI) pendant l' INTUBATION ENDOTRACHÉALE pendant la réanimation s'ils sont effectués À L'EXTÉRIEUR de la salle d'accouchement / salle de travail?

- ☐ Pas de protection spécifique
- ☐ Protection de base (p. Ex. Masque facial, gants et blouse) pour toute réanimation
- ☐ Protection avancée (par exemple, masques faciaux, gants, lunettes / visière faciale et blouse) pour toute réanimation
- ☐ Veuillez préciser s'il y a une différence entre l'accouchement par voie basse et l'accouchement par césarienne ____
- ☐ Autre ____

Dans votre hôpital ou votre unité néonatale, quelles sont les recommandations concernant les équipements de protection individuelle (EPI) pendant l' INTUBATION ENDOTRACHÉALE **pendant la réanimation s'ils sont effectués À L'INTÉRIEUR de la salle**

d'accouchement / de travail (sur une table de réanimation séparée)?

- ☐ Pas de protection spécifique
- ☐ Protection de base (p. Ex. Masque facial, gants et blouse) pour toute réanimation
- ☐ Protection avancée (par exemple, masques faciaux, gants, lunettes / visière faciale et blouse) pour toute réanimation
- ☐ Veuillez spécifier si différent après l'accouchement vaginal ou l'accouchement par césarienne ____
- ☐ Autre ____

End of Block: Delivery room management of baby born to SARS-CoV-2 positive/suspected mothers

---

Start of Block: Admission to neonatal unit of baby born to SARS-CoV-2 positive/suspected mothers

Admission à l'unité néonatale des nourrissons nés de mères séropositives au SRAS-CoV-2 ou soupçonnées d'être positives Définitions: Les procédures génératrices d'aérosols comprennent le traitement par canules nasales haut débit (nHFT / HFNC), la pression positive continue nasale ou PPC nasale (nasal Continuous Positive Airway Pressure, nCPAP), l'intubation et la ventilation mécanique. L'isolement respiratoire de type gouttelettes / contact est défini comme l'utilisation d'un masque chirurgical, d'une blouse et de gants. L'unité néonatale est une unité /salle/service où vous prenez soin des nouveau-nés nécessitant plus que les soins de routine (ex. unité de néonatologie soins courants, soins intensifs, réanimation néonatale).

Quelles sont les précautions d'hygiène standard dans votre unité néonatale lorsqu'il n'y a PAS BESOIN de procédures générant des aérosols?

- [ ] Chambre multiple pour plusieurs patients
- [ ] Chambre seule pour patient unique
- [ ] Chambre à pression négative
- [ ] Ne sais pas
- [ ] Incubateur / couveuse
- [ ] Berceau / lit chauffant
- [ ] Ne sais pas
- [ ] 1 bébé par lit / lit bébé
- [ ] > 1 bébé par lit / lit bébé
- [ ] Ne sais pas
- [ ] Hygiène standard (mains et vêtements propres)
- [ ] Gouttelettes respiratoires / Isolement de contact avec masque facial de base
- [ ] Gouttelettes respiratoires / Isolement par contact avec des masques respiratoires avancés
- [ ] Ne sais pas

Quelles sont les précautions d'hygiène standard dans votre unité de néonatologie lorsqu'un nourrisson BENEFICIE de procédures générant des aérosols?

- ▢ Chambre multiple pour plusieurs patients
- ▢ Chambre seule pour patient unique
- ▢ Chambre à pression négative
- ▢ Ne sais pas
- ▢ Incubateur / couveuse
- ▢ Berceau / lit chauffant
- ▢ Ne sais pas
- ▢ 1 bébé par lit / lit bébé
- ▢ > 1 bébé par lit / lit bébé
- ▢ Ne sais pas
- ▢ Hygiène standard (mains et vêtements propres)
- ▢ Gouttelettes respiratoires / Isolement de contact avec masque facial de base
- ▢ Gouttelettes respiratoires / Isolement par contact avec des masques respiratoires avancés
- ▢ Ne sais pas

End of Block: Admission to neonatal unit of baby born to SARS-CoV-2 positive/suspected mothers

---

Start of Block: Asymptomatic infants born to SARS-CoV-2 positive or suspected positive mothers

Nouveau-nés asymptomatiques nés de mères positives ou suspectes de SARS-CoV-2 ou – séparation/isolement, alimentation et sortie Définitions: Les procédures génératrices d'aérosols comprennent le traitement par canules nasales haut débit (nHFT / HFNC), la pression positive continue nasale ou PPC nasale (nasal Continuous Positive Airway Pressure, nCPAP), l'intubation et la ventilation mécanique. Séparation de la mère et de l'enfant réfère à la séparation physique de la mère et du nouveau-né (interdit d'être dans la même chambre) afin de réduire le risqué potentiel de transmission maternelle au nouveau-né L'unité néonatale est une unité /salle/service où vous prenez soin des nouveau-nés nécessitant plus que les soins de routine (ex. unité de néonatologie soins courants, soins intensifs, réanimation néonatale). L'unité de maternité est une unité / salle de votre hôpital où vous assistez à l'accouchement de nouveau-nés, ou le lieu où les mères et les nouveau-nés restent après l'accouchement (c.-à-d., à la fois les salles de naissance et les unités de suites de couches, maternité, post partum).

OÙ sont pris en charge les nouveau-nés ASYMPTOMATIQUES nés de mères positives ou suspectes de SARS-CoV-2 INITIALEMENT APRÉS LA NAISSANCE?

- ☐ Restent avec la mère comme cela se faisait de routine auparavant (pas de chambre individuelle disponible)
- ☐ Restent avec la mère dans une chambre individuelle, comme cela se faisait habituellement auparavant
- ☐ Restent avec la mère dans une chambre individuelle, mais dans un berceau / incubateur/couveuse séparé d'une certaine distance de la mère (avec ou sans barrière physique comme un rideau)
- ☐ Restent dans une chambre séparée de la mère à l'hôpital
- ☐ Autre ____
- ☐ Ne sais pas

Le père, le partenaire ou un autre membre de la famille peut-il rester auprès de nourrissons ASYMPTOMATIQUES dans le lieu que vous avez indiqué précédemment?

- ☐ Oui, sans restrictions
- ☐ Oui, avec des restrictions de temps
- ☐ Non
- ☐ Ne sais pas
- ☐ Uniquement si le père / partenaire / membre de la famille est testé négatif
- ☐ Autre commentaire ____

Page Break

Pour les nourrissons ASYMPTOMATIQUES dont les mères sont positives ou suspectes de SARS-CoV-2: Dans notre unité de maternité / néonatologie, nous suivons les souhaits de la mère / famille quant à savoir si leur enfant restera avec la mère après la naissance.

- ☐ Pas du tout d'accord
- ☐ Pas d'accord
- ☐ Ni d'accord ni en désaccord
- ☐ D'accord
- ☐ Tout à fait d'accord
- ☐ Ne sais pas

Alimentation des nourrissons ASYMPTOMATIQUES nés de mères positives ou suspectes de SARS-CoV-2

- ☐ Recommandons l'allaitement maternel
- ☐ Ne recommandons pas l'allaitement maternel, mais conseillons à la mère d'exprimer/tirer le lait
- ☐ Recommandons le lait artificiel/préparation pour nourrissons
- ☐ Ne sais pas

Pour les nourrissons ASYMPTOMATIQUES de mères positives ou suspectes de SARS-CoV-2: Dans notre unité de maternité / néonatologie, nous suivons les souhaits de la mère / famille concernant l'allaitement.

- ☐ Pas du tout d'accord
- ☐ Pas d'accord
- ☐ Ni d'accord ni en désaccord
- ☐ D'accord
- ☐ Tout à fait d'accord
- ☐ Ne sais pas

Pour ceux qui recommandent l'allaitement maternel chez les mères positives ou suspectes de SARS-CoV-2, quel sont vos CONSEILS D'HYGIÈNE pour les mères? (Cochez toutes les cases)

- ☐ Se laver les mains avec du savon et de l'eau avant de nourrir
- ☐ Laver le sein avant d'allaiter
- ☐ Utilisation d'un couvre-visage, y compris un masque ou un foulard, pendant l'allaitement

---

QUAND autorisez-vous la sortie d'hospitalisation des nouveau-nés ASYMPTOMATIQUES? (en supposant que les mères sont positives ou suspectes de SARS-SoV-2, mais dans un état clinique stable permettant d'autoriser la sortie)

- ▢ Le moment de la sortie n'est pas différent de la routine antérieure
- ▢ Recommandation de rester plus longtemps à l'hôpital pour observation
- ▢ Recommandation de quitter plus tôt pour éviter une éventuelle transmission à d'autres patients / personnel
- ▢ Ne sais pas
- ▢ Autre ____

End of Block: Asymptomatic infants born to SARS-CoV-2 positive or suspected positive mothers

---

Start of Block: Viral testing and laboratory analyses

Tests viraux et analyses de laboratoire

Des tests viraux sur écouvillons nasopharyngés sont-ils disponibles?

- ○ Indisponible
- ○ Accès disponible mais intermittent
- ○ Disponible, sans restrictions importantes
- ○ Ne sais pas

Dans quels délais les résultats des tests viraux sont-ils disponibles?

- ☐ <6 heures
- ☐ 6-24 heures
- ☐ 24-72 heures
- ☐ > 72 heures
- ☐ Ne sais pas
- ☐ Autre ____

La réalisation du test viral dépend-elle de la capacité de la famille à payer le test?

- ○ Oui ____
- ○ Non ____
- ○ Ne sais pas

Page Break

En plus des écouvillons nasopharyngés, sélectionnez tout test supplémentaire que vous effectuez de façon routinière chez les nourrissons né de mères positives ou suspectes de SARS-CoV-2?

- ☐ Test sanguin virologique; PCR
- ☐ Sérologie sanguine
- ☐ Tabouret
- ☐ Analyse du LCR
- ☐ Placenta
- ☐ Liquide amniotique
- ☐ ⊗Aucun

Vous avez sélectionné « Aucun. » Avez-vous des commentaires additionnels sur le dépistage? (facultatif)

____

____

____

____

____

End of Block: Viral testing and laboratory analyses

Start of Block: Promising ideas (i.e., with potential improvement or benefit) in neonatal unit

Idées prometteuses (c.-à-d. avec amélioration ou avantage potentiels) pour les soins cliniques, le soutien et les familles

Veuillez décrire brièvement si vous avez mis en œuvre de nouvelles idées prometteuses de SOINS CLINIQUES pendant la pandémie de COVID-19 (optionnel).

Veuillez décrire brièvement si vous avez mis en œuvre de nouvelles idées prometteuses pour SOUTENIR LOCALEMENT LES SOIGNANTS EN NEONATOLOGIE pendant la pandémie de COVID-19 (optionnel).

Veuillez décrire brièvement si vous avez mis en œuvre de nouvelles idées prometteuses de SOUTIEN FAMILIAL / PARENTAL pendant la pandémie de COVID-19 (optionnel).
