# Supplementary material for: COVID-19 preparedness—a survey among neonatal care providers in low- and middle-income countries
Source: J Perinatol. 2021 Apr 13;41(5):988–97. doi: 10.1038/s41372-021-01019-4 (PMC8042838; doi:10.1038/s41372-021-01019-4)
Supplement: Supplementary file 3 — Survey (Spanish) [file 41372_2021_1019_MOESM3_ESM.pdf]

COVID-19 Neonatal Guidelines Survey - Spanish

Estimado Colega

El COVID-19 es una catástrofe global que también nos afecta a quienes trabajamos en la atención perinatal y neonatal.

Afortunadamente, los recién nacidos no parecen pertenecer al grupo de mayor riesgo de infecciones graves por COVID-19, pero debido a que tienen un sistema inmune inmaduro, debemos de ser cuidadosos al cuidar a esta población tan vulnerable.

La OMS y otras asociaciones médicas han publicado directrices y guías sobre cómo manejar a los recién nacidos que nacen de madres positivas con COVID-19.

El objetivo de esta encuesta es identificar las pautas y guías de preparación para COVID-19 entre los profesionales de atención médica neonatal en todo el mundo, incluyendo en comunidades de bajos recursos en donde nacen la mayoría de los recién nacidos. Estos resultados podían ayudar a enfocarnos especialmente en entornos en donde la comunidad global necesita asignar más enfoque y recursos. **Por favor responda en cuanto al estado ACTUAL en su hospital. Si hay alguna pregunta que sea "muy delicada", por favor elija "No sé".**

Nuestro objetivo es publicar los resultados de esta encuesta en una revista médica internacional.

Todos los que respondan a esta encuesta serán mencionados como "colaboradores internacionales" en la próxima publicación. Todos los datos se manejarán con cuidado para no identificar datos personales u otros datos sensibles. El manejo de datos ha sido aprobado por el comité de revisión institucional (IRB) de la Facultad de Medicina de la Universidad de Stanford, en Estados Unidos.

Apreciamos mucho su participación. La encuesta en línea tomará aproximadamente 20 minutos de su tiempo en completarse.

¡Muchas gracias de antemano por participar!

Grupo coordinador: Claus Klingenberg, Anna Lavizzari, Sithembiso Velaphi, Omer Erdeve, Hon Kin Cheong, Surender Bisht, Irfan Waheed, Alex Stevenson, Nisreen Al-Kafi, Nestor Vain, Gonzalo Mariani, Victor Javier Lara Diaz, John Zupancic, Charles Christoph Roehr, Danielle Ehret y Jochen Profit

Indique su nombre si desea ser reconocido como "Colaborador internacional" (opcional)

____

Proporcione su dirección de correo electrónico si desea ser reconocido como un "colaborador internacional" (opcional)

____

¿En qué país se encuentra su hospital?

____

¿En qué ciudad se encuentra su hospital?

____

¿Cómo se llama su hospital?

____
____
____
____
____

Page Break

Por favor lea las siguientes definiciones:

Inmediatamente después del nacimiento se desconoce el estado de SARS-CoV-2 del bebé. Hemos definido lo siguiente en estos puntos:

SARS-CoV-2 es el nombre del virus que causa COVID-19 **El equipo de protección personal (EPP)** incluye guantes, batas o camisolines, mascarillas o barbijos, desinfectante

Los procedimientos de generación de aerosoles incluyen cánula nasal de alto flujo (CNAF), presión nasal positiva continua en las vías respiratorias (nCPAP), intubación y ventilación mecánica. **Aislamiento** significa que se están tomando medidas para reducir el contacto con otros bebés y reducir el contacto con el personal de salud. **Separación de la madre** significa que la madre y el bebe están separados físicamente (no se les permite mantener al bebé con ella / en la misma habitación) para reducir el riesgo potencial de transmisión postnatal de la madre al bebé. **El aislamiento de gotas y de contacto respiratorio** se define como el uso de una mascarilla/barbijo quirúrgico, bata/camisolín y guantes. **Las mascarillas/barbijos avanzados** incluyen aquellos designados N95 / FFP2 / FFP3 **La unidad neonatal** es cualquier unidad / sala donde se atiende a recién nacidos que requieren MÁS que atención de RUTINA (por ejemplo, unidad de cuidados intensivos, unidad de cuidados intermedios, etc.) **La unidad de maternidad** es una unidad / sala en su hospital donde se atienden partos de recién nacidos, o donde las madres y los recién nacidos permanecen juntos después del parto (es decir, incluye **AMBAS** unidades obstétricas y postparto)

End of Block: Introduction

Start of Block: Background data

Antecedentes y epidemiología local
Definición:
La unidad neonatal es cualquier unidad / sala donde se atiende a recién nacidos que requieren MÁS que atención de RUTINA (por ejemplo, unidad de cuidados intensivos, unidad de cuidados intermedios, etc.)

Número anual de nacimientos en su hospital
(Por favor, indique un rango estimado)

- 0
- 1-1000
- 1001-3000
- 3001-6000
- > 6000

Número anual de pacientes ingresados a la unidad neonatal en su hospital
(Por favor, indique un rango estimado)

- 1-100
- 101-500
- 501-1000
- 1001-2000
- > 2000

¿Cuál es el peso al nacer (en gramos) más bajo que cuida HABITUALMENTE en su hospital? (Por favor, indique un rango estimado)

- <751
- 751-1000
- 1001-1500
- 1501-2000
- > 2000

Recursos locales en su unidad neonatal - soporte respiratorio

○ Podemos proporcionar ventilación mecánica invasiva y no invasiva

○ Podemos proporcionar asistencia respiratoria no invasiva (p. ej., Presión positiva continua en las vías respiratorias (CPAP), cánula nasal de alto flujo (HFNC), pero no ventilación mecánica)

○ Solo podemos proporcionar oxígeno suplementario

Page Break

Aproximadamente, ¿qué proporción de sus pacientes se encuentran en el cuarto MÁS BAJO del rango de ingresos económicos de su país?

○ <25%

○ 25-75%

○ > 75%

○ No se

Su hospital es:

○ **PÚBLICO**: Las instalaciones bajo jurisdicción o financiamiento del gobierno nacional o local o militar (por ejemplo, hospital del gobierno, centro de salud, hogar de maternidad del gobierno, otra institución pública)

○ **PRIVADO, con fines de lucro**: Las instalaciones son entidades comerciales bien definidas y con fines de lucro (por ejemplo, hospital privado con fines de lucro, hogar de maternidad privado con fines de lucro u otro centro privado con fines de lucro)

○ **PRIVADO, SIN fines de lucro**: Las instalaciones son entidades comerciales bien definidas, pero SIN fines de lucro (por ejemplo, hospital sin fines de lucro, organización no gubernamental, hospital de fundación o misión, hogar de maternidad fines de lucro, otra instalación privada sin fines de lucro)

○ Otro ____

○ No se

Su hospital es:

○ Un centro de referencia regional

○ Un hospital comunitario local (no recibe derivaciones externas)

○ Otro ____

○ No se

La preparación y atención de servicios por COVID-19 ha sido una carga económica para mi HOSPITAL

- Totalmente en desacuerdo
- Un poco en desacuerdo
- Ni de acuerdo ni en desacuerdo
- Un poco de acuerdo
- Totalmente de acuerdo
- No se

En mi hospital, la atención de COVID-19 es una carga económica para los PACIENTES

- Totalmente en desacuerdo
- Un poco en desacuerdo
- Ni de acuerdo ni en desacuerdo
- Un poco de acuerdo
- Totalmente de acuerdo
- No se

En nuestra unidad neonatal durante la pandemia de COVID-19

- ☐ Hemos experimentado un déficit de personal de enfermería
- ☐ Hemos experimentado un déficit de personal médico
- ☐ Hemos experimentado un déficit de AMBOS, personal de enfermería y médico.
- ☐ NO hemos experimentado un déficit de personal de enfermería o médico
- ☐ No se
- ☐ Comentario Adicional: ____

Page Break

End of Block: Background data

Start of Block: General Questions related to COVID-19

Preguntas generales relacionadas con COVID-19

Definición:

La unidad neonatal es cualquier unidad / sala donde se atiende a recién nacidos que requieren MÁS que atención de RUTINA (por ejemplo, unidad de cuidados intensivos, unidad de cuidados intermedios, etc.)

En mi unidad neonatal seguimos las siguientes pautas para el cuidado de pacientes con sospecha o confirmación de SARS-CoV-2: (marque todas las que aplique)

- [ ] Guías de la OMS
- [ ] Pautas/guías nacionales
- [ ] Pautas/guías locales / hospitalarias
- [ ] ⊗ Ninguna
- [ ] Comentario Adicional: ____

Responda las siguientes preguntas en relación con su unidad neonatal:

	Totalmente en desacuerdo	Un poco en desacuerdo	Ni de acuerdo ni en desacuerdo	Un poco de acuerdo	Totalmente de acuerdo	***No se***
Las guías relacionadas con COVID-19 están accesibles para los profesionales de la salud (por ejemplo, copia impresa, sitio web local, etc.)	○	○	○	○	○	○
Los profesionales de la salud han recibido capacitación específica para el uso de nuestras guías de COVID-19	○	○	○	○	○	○
Los profesionales de la salud en su mayoría siguen nuestras guías de COVID-19	○	○	○	○	○	○

Seleccionó "Ninguno". ¿Tiene algún comentario adicional sobre las guías/ recomendaciones para el cuidado de pacientes con sospecha o confirmación de SARS-CoV-2?(opcional)

____

____

Page Break

A las MADRES positivas o con sospecha de ser positivas con SARS-CoV-2 se les aconseja ir a parir a centros de referencia específicos para COVID-19 en mi región / país.

- Si siempre
- Sí, cuando sea posible
- No
- No se
- Otros (especificar) ____

LOS RECIÉN NACIDOS positivos o con sospecha de ser positivos con SARS-CoV-2 se trasladan a centros de referencia específicos para COVID-19 en mi región / país.

- Si siempre
- Sí, cuando sea posible
- No
- No se
- Otros (especificar) ____

Page Break

Tengo la impresión de que la pandemia de COVID-19 ha aumentado la mortalidad de otros RECIÉN NACIDOS NO INFECTADOS POR COVID-19 en mi unidad neonatal.

- ○ Si
- ○ No
- ○ No lo se

La tasa de admisión a mi unidad neonatal durante la pandemia de COVID-19 ha:

- ○ Aumentado
- ○ Disminuido
- ○ No ha cambiado significativamente
- ○ No lo se

Las razones del aumento en la mortalidad en INFANTES NO-INFECTADOS CON COVID-19 en mi unidad neonatal incluyen
(marque todas las que correspondan):

- ☐ Escasez de personal
- ☐ Falta de equipamiento
- ☐ Falta de equipo de protección personal (EPP; guantes, batas, máscaras/cubre bocas, desinfectante)
- ☐ ⊗ No se
- ☐ Comentarios adicionales

____

Las razones de la **disminución** en la tasa de admisión incluyen (marque todas las que correspondan):

- ☐ Disminución de partos en mi hospital; madres tratando de evitar la exposición a COVID-19
- ☐ Disminución de partos en mi hospital; por restricciones de viaje para madres
- ☐ Disminución en los traslados neonatales; por evitar la exposición de recién nacidos a COVID-19
- ☐ Disminución en los traslados neonatales; por restricciones de viaje para familias
- ☐ Disminución en los traslados neonatales; por escasez de ambulancias o de personal
- ☐ Otro ____

Page Break

End of Block: General Questions related to COVID-19

Start of Block: Isolation, resources, and visitor restrictions in your neonatal unit

Medidas de aislamiento y recursos en su unidad neonatal.

Definiciones:

El aislamiento de gotas y de contacto respiratorio se define como el uso de una mascarilla/barbijo quirúrgico, bata/camisolín y guantes.

La unidad neonatal es cualquier unidad / sala donde se atiende a recién nacidos que requieren MÁS que atención de RUTINA (por ejemplo, unidad de cuidados intensivos, unidad de cuidados intermedios, etc.)

Posibilidades de aislamiento en su unidad neonatal:
(marque todo lo que corresponda):

- ▢ No hay habitaciones individuales adicionales (todas las habitaciones en la unidad neonatal son compartidas (para múltiples pacientes)
- ▢ Número limitado de habitaciones individuales.
- ▢ Las habitaciones individuales son suficientes
- ▢ No se
- ▢ Otros comentarios ____

¿Cuáles de los siguientes recursos están disponibles en su unidad neonatal?
(marque todo lo que corresponda):

- [ ] Agua potable
- [ ] Jabón
- [ ] Toallas limpias
- [ ] Guantes médicos
- [ ] Mascarillas/barbijos básicos
- [ ] Mascarillas/barbijos avanzados (incluidas las designadas N95 / FFP2 / FFP3)
- [ ] Batas/camisolines desechables (de un solo uso)
- [ ] ⊗ Ninguno
- [ ] Comentarios adicionales al equipo de protección personal disponible

____

Seleccionó "Ninguno". ¿Tiene algún comentario adicional sobre la disponibilidad de equipamiento?
(opcional)

____

____

____

____

____

Comente qué porcentaje de tiempo tiene acceso a cada recurso en su unidad neonatal:

	0%	menos de 50%	mas de 50%	100%
Agua potable	○	○	○	○
Jabón	○	○	○	○
Toallas limpias	○	○	○	○
Guantes médicos para el cuidado de recién nacidos sospechosos / positivos del SARS-CoV-2.	○	○	○	○
Mascarillas/barbijos básicos para el cuidado de recién nacidos sospechosos / positivos de SARS-CoV-2.	○	○	○	○
Mascarillas/barbijos avanzados para el cuidado de recién nacidos sospechosos / positivos del SARS-CoV-2 y posibles procedimientos de generación de aerosoles.	○	○	○	○
Batas/ camisolines desechables de un solo uso cuando se atiende a recién nacidos sospechosos / positivos del SARS-CoV-2.	○	○	○	○

Page Break

Restricciones para visitantes en su unidad neonatal

¿Ha implementado restricciones GENERALES de visitantes durante la actual pandemia de COVID-19 en su unidad neonatal?

- ☐ ⊗ No hay restricciones adicionales
- ☐ ⊗ No se permiten visitas
- ☐ Restricción del número de visitantes.
- ☐ Solo se permite la visita de la madre y el padre
- ☐ Solo se permite la visita de la madre o el padre pero de a uno por vez
- ☐ Restricciones para personas que no son de la familia.
- ☐ Restricción a la duración de la visita.
- ☐ Otros (por favor especifique)

____

Seleccionó "Sin restricciones adicionales". ¿Tiene algún comentario adicional sobre las restricciones de visitantes durante la actual pandemia de COVID-19 en su unidad neonatal? (opcional)

____

____

____

____

____

End of Block: Isolation, resources, and visitor restrictions in your neonatal unit

---

Start of Block: Delivery room management of baby born to SARS-CoV-2 positive/suspected mothers

Manejo de bebés nacidos de madres positivas o con sospecha de SARS-CoV-2 en la sala de partos
Definiciones: La unidad de maternidad es una unidad / sala en su hospital donde se atienden nacimientos, o donde las madres y los recién nacidos permanecen juntos después del parto (es decir, incluye **AMBAS** unidades obstétricas y posparto) La unidad neonatal es cualquier unidad / sala donde se atiende a recién nacidos que requieren **MÁS** que atención de **RUTINA** (por ejemplo, unidad de cuidados intensivos, unidad de cuidados intermedios, etc.)

Mi hospital tiene una sala / unidad de maternidad.

○ Si

○ No hay partos en mi hospital

En su hospital o unidad neonatal, ¿cuáles son las recomendaciones con respecto al PINZAMIENTO Y CORTE DE CORDÓN UMBILICAL en bebés nacidos de madres positivas o con sospecha de SARS CoV-2?

▢ Inmediato

▢ Diferido/ demorado

▢ No se

▢ Otros (especificar) ____

En su hospital o unidad neonatal, durante la reanimación neonatal AFUERA de la sala de partos, ¿Cuáles son las recomendaciones en cuanto al equipo de protección personal (EPP) para LA VENTILACIÓN CON BOLSA Y MÁSCARA?

- [ ] Sin protección específica
- [ ] Protección básica (p. ej., Mascarilla/barbijo, guantes y bata/ camisolín) para toda reanimación
- [ ] Protección avanzada (p. ej., Mascarillas/ barbijo, guantes, gafas/ antiparras/ máscara facial transparente y bata/ camisolín) para toda reanimación
- [ ] Especifique si es diferente de acuerdo con la vía de parto: vaginal o cesárea

____

- [ ] Comentarios adicionales

____

En su hospital o unidad neonatal, durante la reanimación neonatal DENTRO de la sala de partos, ¿Cuáles son las recomendaciones en cuanto al equipo de protección personal (EPP) para LA VENTILACIÓN CON BOLSA Y MÁSCARA?

- [ ] No hay protección específica si la reanimación se realiza DENTRO de la sala de parto (en una mesa de reanimación separada)
- [ ] Protección básica (p. ej., Mascarilla/ barbijo, guantes y bata/ camisolín) para toda reanimación
- [ ] Protección avanzada (p. ej., Mascarillas/ barbijo, guantes, gafas/ antiparras/ máscara facial transparente y bata/ camisolín) para toda reanimación
- [ ] Especifique si es diferente de acuerdo con la vía de parto: vaginal o cesárea

____

- [ ] Comentarios adicionales

____

Page Break

En su hospital o unidad neonatal, durante la reanimación neonatal AFUERA de la sala de partos, ¿Cuáles son las recomendaciones en cuanto al equipo de protección personal (EPP) durante LA INTUBACIÓN ENDO-TRAQUEAL?

- ☐ Sin protección específica
- ☐ Mascarilla/ barbijo de protección básica, guantes y bata/ camisolín para toda reanimación
- ☐ Mascarillas/ barbijos avanzadas, guantes, gafas/ antiparras/ máscara facial transparente y bata/ camisolín para toda la reanimación
- ☐ Especifique si es diferente de acuerdo con la vía de parto: vaginal o cesárea
____
- ☐ Comentarios adicionales
____

En su hospital o unidad neonatal, ¿cuáles son las recomendaciones con respecto al Equipo de protección personal (EPP) durante reanimación neonatal cuando se necesita hacer una

INTUBACIÓN ENDO-TRAQUEAL DENTRO de la sala de partos (en una mesa de reanimación separada)?

- ▢ Sin protección específica
- ▢ Mascarilla/ barbijo de protección básica, guantes y bata/ camisolín para toda reanimación
- ▢ Mascarillas/ barbijos avanzadas, guantes, gafas/ antiparras/ máscara facial transparente y bata/ camisolín para toda la reanimación
- ▢ Especifique si es diferente después del parto vaginal o cesárea ____
- ▢ Comentarios adicionales ____

End of Block: Delivery room management of baby born to SARS-CoV-2 positive/suspected mothers

---

Start of Block: Admission to neonatal unit of baby born to SARS-CoV-2 positive/suspected mothers

Admisión a la unidad neonatal de bebés nacidos de madres positivas o con sospecha de infección de SARS-CoV-2 Definiciones: Los procedimientos de generación de aerosoles incluyen cánula nasal de alto flujo (CNAF), presión nasal positiva continua en las vías respiratorias (CPAPn), intubación y ventilación mecánica. El aislamiento de gotas / contacto respiratorio se define como el uso de una mascarilla/ barbijo quirúrgico, bata/ camisolín y guantes. La unidad neonatal es cualquier unidad / sala donde se atiende a recién nacidos que requieren MÁS que atención de RUTINA (por ejemplo, unidad de cuidados intensivos, unidad de cuidados intermedios, etc.)

¿Cuáles son las medidas de higiene y precauciones de rutina en su unidad neonatal cuando NO HAY NECESIDAD de procedimientos generadores de aerosoles?

- [ ] Habitación compartida (múltiples pacientes)
- [ ] Habitación individual
- [ ] Habitación con presión negativa
- [ ] No se
- [ ] Incubadora
- [ ] Cuna con calentador radiante/ Servocuna
- [ ] No se
- [ ] 1 bebé por cama / cuna
- [ ] > 1 bebé por cama / cuna
- [ ] No se
- [ ] Higiene estándar (manos y ropa limpias)
- [ ] Aislamiento de gotas y aislamiento de contacto respiratorio con mascarilla/ barbijo básico
- [ ] Aislamiento de gotas y aislamiento de contacto respiratorio con mascarilla/ barbijo facial avanzado
- [ ] No se

¿Cuáles son las medidas de higiene y precauciones de rutina en su unidad neonatal cuando UN BEBÉ NECESITA procedimientos generadores de aerosoles?

- ☐ Habitación compartida (múltiples pacientes)
- ☐ Habitación individual
- ☐ Habitación con presión negativa
- ☐ No se
- ☐ Incubadora
- ☐ Cuna con calentador radiante/ Servocuna
- ☐ No se
- ☐ 1 bebé por cama / cuna
- ☐ > 1 bebé por cama / cuna
- ☐ No se
- ☐ Higiene estándar (manos y ropa limpias)
- ☐ Aislamiento de gotas y aislamiento de contacto respiratorio con mascarilla/ barbijo básico
- ☐ Aislamiento de gotas y aislamiento de contacto respiratorio con mascarilla/ barbijo facial avanzado
- ☐ No se

End of Block: Admission to neonatal unit of baby born to SARS-CoV-2 positive/suspected mothers

Start of Block: Asymptomatic infants born to SARS-CoV-2 positive or suspected positive mothers

Bebés asintomáticos nacidos de madres positivas o con sospecha de SARS-CoV-2: Separación, alimentación y alta del hospital

Definiciones:

Los procedimientos de generación de aerosoles incluyen cánula nasal de alto flujo (CNAF), presión nasal positiva continua en las vías respiratorias (CPAPn), intubación y ventilación mecánica.

Separación de la madre significa que la madre y el bebe están separados físicamente (no se les permite mantener al bebé con ella / en la misma habitación) para reducir el riesgo potencial de transmisión postnatal de la madre al bebé.

La unidad neonatal es cualquier unidad / sala donde se atiende a recién nacidos que requieren MÁS que atención de RUTINA (por ejemplo, unidad de cuidados intensivos, unidad de cuidados intermedios, etc.)

La unidad de maternidad es una unidad / sala en su hospital donde se atienden nacimientos, o donde las madres y los recién nacidos juntos permanecen después del parto (es decir, incluye AMBAS unidades obstétricas y posparto)

¿DÓNDE se les da atención a los recién nacidos ASINTOMÁTICOS hijos de madres con sospecha o confirmación de SARS-CoV-2, INICIALMENTE DESPUES DEL PARTO?

- [ ] Se quedan con la madre como se hacía habitualmente (no hay habitación individual disponible)
- [ ] Se quedan con la madre en una habitación individual, como se hacía habitualmente
- [ ] Se quedan con la madre en una habitación individual, pero en una cuna /incubadora separada una cierta distancia de la madre (con o sin barrera física, como una cortina)
- [ ] Se queda en una habitación separados de la madre en el hospital
- [ ] Otro ____
- [ ] No se

¿Al padre, la pareja o algún otro miembro de la familia se le permite permanecer con bebés ASINTOMÁTICOS en el tipo de habitación que se indico en la pregunta anterior?

- [ ] Si, sin restricciones
- [ ] Sí, con restricciones de tiempo.
- [ ] No
- [ ] No lo se
- [ ] Solo si el padre / pareja / miembro de la familia dio negativo
- [ ] Otro comentario ____

Page Break

Para bebes ASINTOMÁTICOS con madres positivas o con sospecha de SARS-CoV-2 positivo: En nuestra unidad de maternidad / neonatal, seguimos los deseos de la madre / familia con respecto a si su bebé permanecerá con la madre después del nacimiento.

- ☐ Totalmente en desacuerdo
- ☐ Un poco en desacuerdo
- ☐ Ni de acuerdo ni en desacuerdo
- ☐ Un poco de acuerdo
- ☐ Totalmente de acuerdo
- ☐ No lo se

Alimentación de bebes ASINTOMÁTICOS nacidos de madres positivas o con sospecha de SARS-CoV-2 positivo

- ☐ Se recomienda amamantar
- ☐ No se recomienda amamantar, pero se le aconseja a la madre extraer la leche
- ☐ Se recomienda usar fórmula
- ☐ No lo se

Para bebes ASINTOMÁTICOS con madres positivas o con sospecha de SARS-CoV-2 positivo: En nuestra unidad de maternidad / neonatal, seguimos los deseos de la madre / familia con respecto a la lactancia materna.

- [ ] Totalmente en desacuerdo
- [ ] Un poco en desacuerdo
- [ ] Ni de acuerdo ni en desacuerdo
- [ ] Un poco de acuerdo
- [ ] Totalmente de acuerdo
- [ ] No lo se

Para quienes recomiendan lactancia materna en madres positivas o con sospecha de SARS-CoV-2 positivo, ¿Qué se les ACONSEJA a las madres en cuanto a medidas de HIGIENE? (Marque todas las que correspondan)

- [ ] Lavado de manos con agua y jabón antes de alimentar
- [ ] Lavado de senos antes de alimentar
- [ ] Uso de cobertura facial, incluyendo mascarilla/ barbijo o bufanda, durante la lactancia.

¿CUÁNDO se les da de alta a bebes ASINTOMÁTICOS? (suponiendo que las madres son positivas o tienen sospecha de SARS-CoV-2 positivo, pero se encuentran en una condición clínica estable para dar el alta)

- ☐ El momento del alta no es diferente de la práctica anterior
- ☐ Se recomienda permanecer más tiempo en el hospital para observación
- ☐ Se recomienda dar de alta antes para evitar la posible transmisión a otros pacientes / personal
- ☐ No se
- ☐ Comentarios adicionales ____

End of Block: Asymptomatic infants born to SARS-CoV-2 positive or suspected positive mothers

---

Start of Block: Viral testing and laboratory analyses

Pruebas virales y análisis de laboratorio.

¿Están disponibles las pruebas virales con hisopos nasofaríngeos?

- ○ No disponible
- ○ Disponible, pero acceso intermitente
- ○ Disponible, sin restricciones significativas
- ○ No lo se

¿Qué tan rápido están disponibles los resultados de las pruebas virales?

- ☐ <6 horas
- ☐ 6-24 horas
- ☐ 24-72 horas
- ☐ > 72 horas
- ☐ No lo se
- ☐ Otro ____

¿Las pruebas virales dependen de la capacidad de la familia para pagar las pruebas?

- ○ Si ____
- ○ No ____
- ○ No se

Page Break

¿Además de los hisopos nasofaríngeos, seleccione cualquier prueba adicional que se realice rutinariamente en bebés nacidos de madres positivas o con sospecha de SARS-CoV-2 positivo?

- ☐ Análisis de sangre viral; PCR
- ☐ Anticuerpos serológicos de sangre
- ☐ Heces
- ☐ Liquido cefalorraquídeo (LCR)
- ☐ Placenta
- ☐ Líquido amniótico
- ☐ ⊗Ninguna

Seleccionó "Ninguno". ¿Tiene algún comentario adicional sobre las pruebas diagnósticas? (opcional)

____

____

____

____

____

End of Block: Viral testing and laboratory analyses

Start of Block: Promising ideas (i.e., with potential improvement or benefit) in neonatal unit

Ideas prometedoras (es decir, con posible mejora o beneficio) para la atención clínica, el apoyo y las familias.

Describa brevemente si ha implementado nuevas ideas prometedoras para el CUIDADO CLÍNICO durante la pandemia de COVID-19 (Opcional).

Describa brevemente si ha implementado nuevas ideas prometedoras para APOYAR A LOS PROFESIONALES LOCALES DE ATENCIÓN MÉDICA NEONATAL durante la pandemia de COVID-19 (Opcional).

Describa brevemente si ha implementado nuevas ideas prometedoras para el APOYO A LOS FAMILIARES / PADRES durante la pandemia de COVID-19 (Opcional).
