# Supplementary figures and images for: COVID-19 preparedness—a survey among neonatal care providers in low- and middle-income countries
Source: J Perinatol. 2021 Apr 13;41(5):988–97. doi: 10.1038/s41372-021-01019-4 (PMC8042838; doi:10.1038/s41372-021-01019-4)

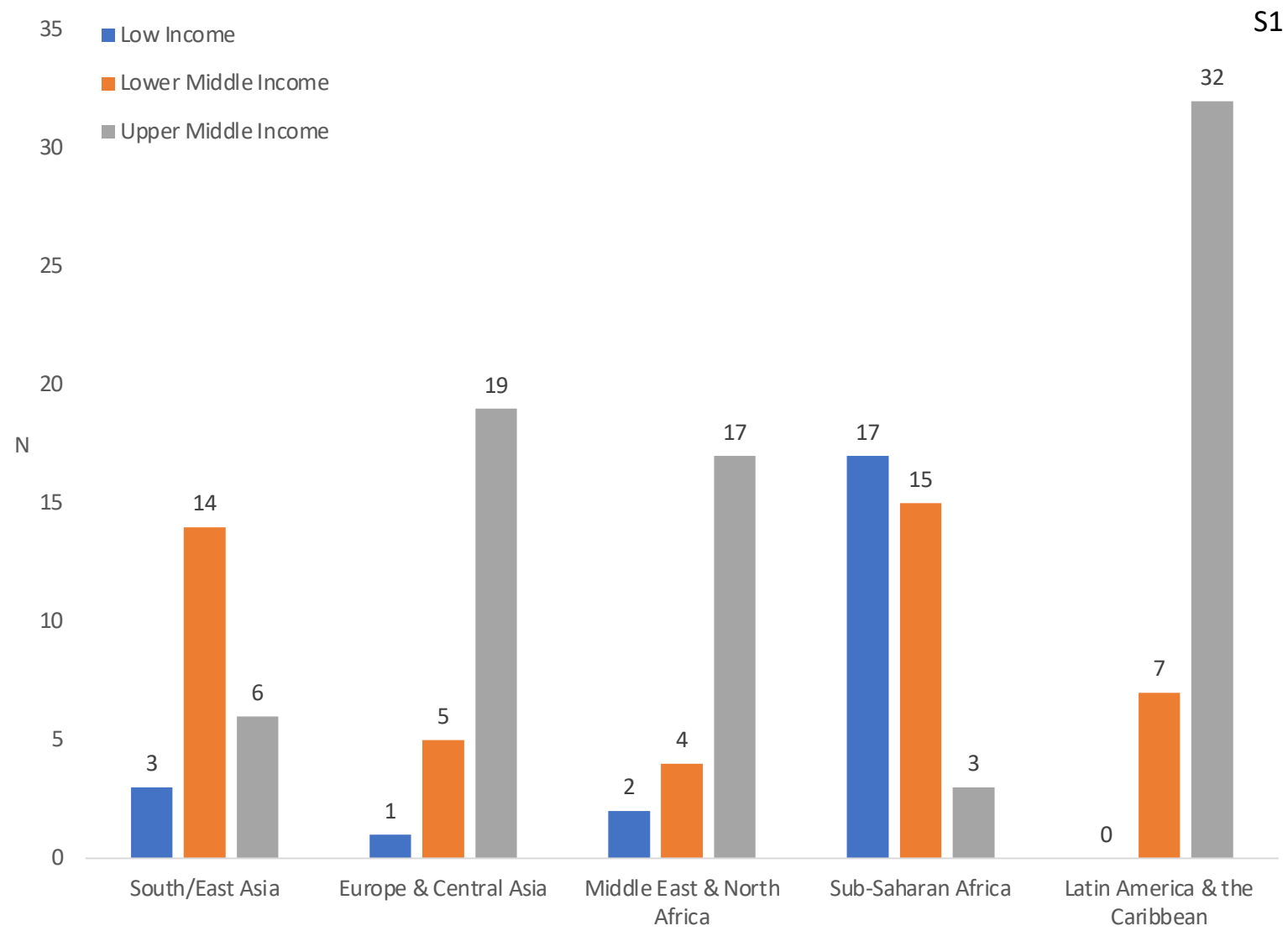

Classification of 145 responses by income level of countries in the five global regions

Supplement: Supplementary file 4 — Supplementary Figure 1 [file 41372_2021_1019_MOESM4_ESM.pdf]

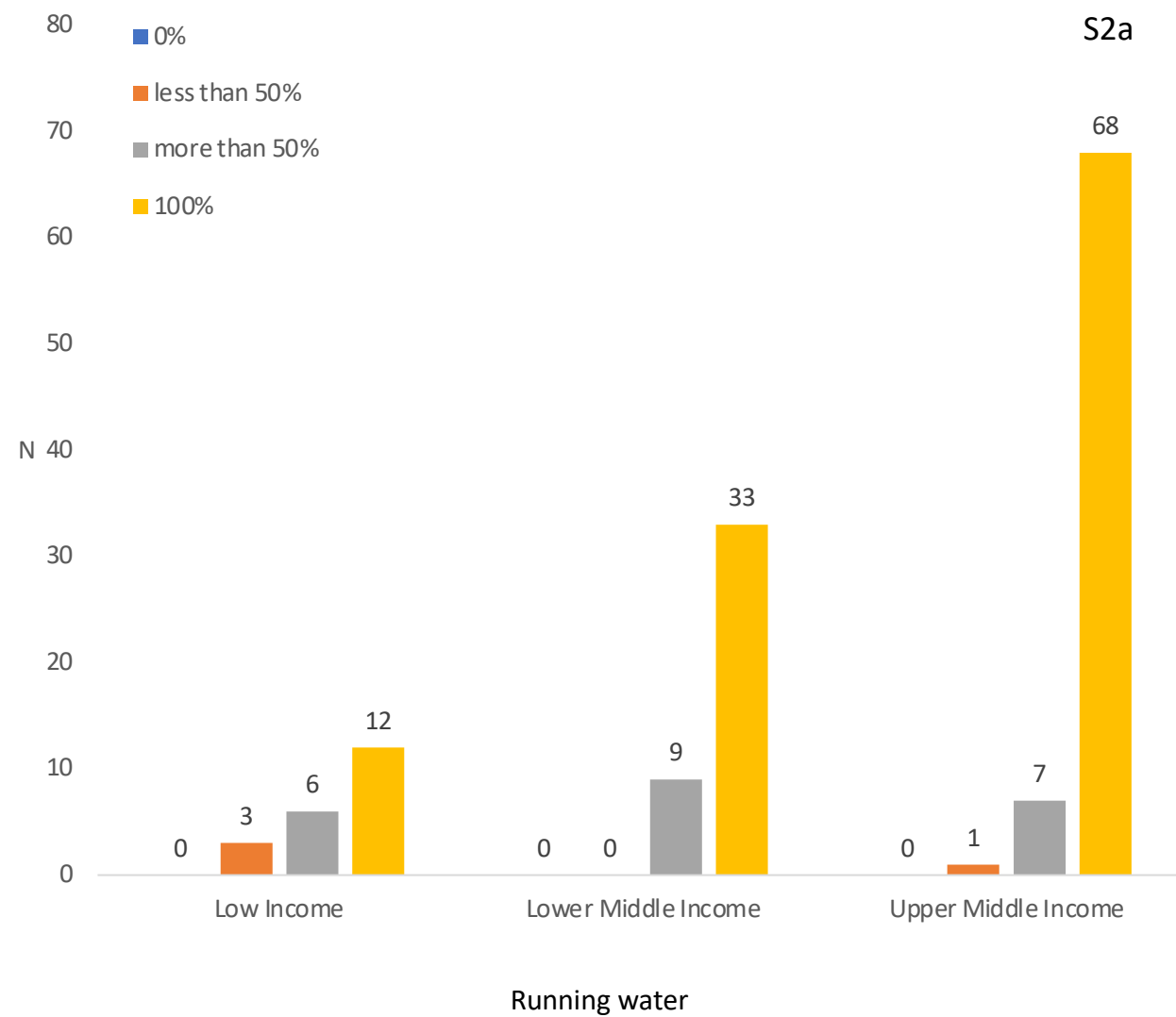

Supplement: Supplementary file 5 — Supplementary Figure 2a [file 41372_2021_1019_MOESM5_ESM.pdf]

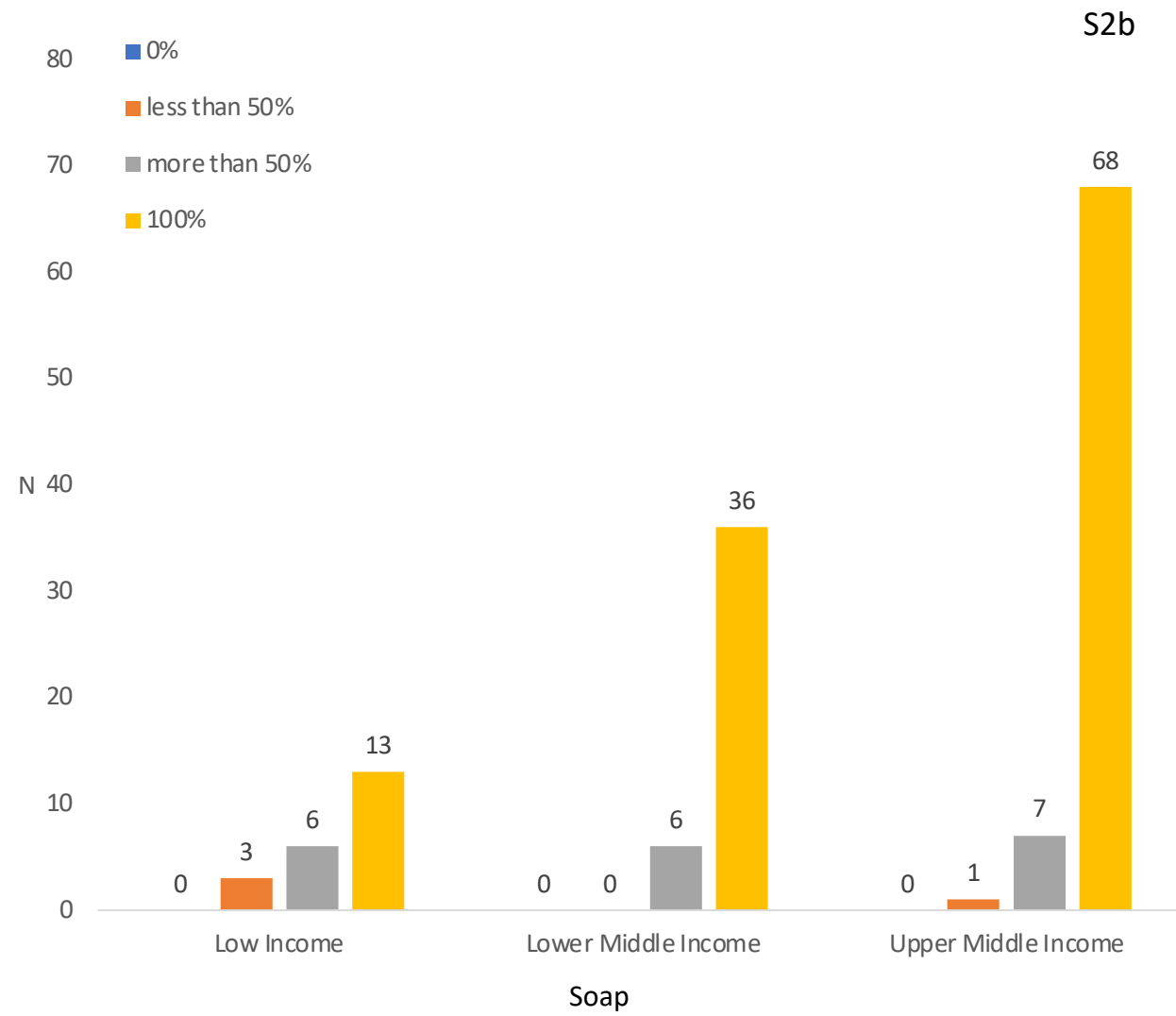

Supplement: Supplementary file 6 — Supplementary Figure 2b [file 41372_2021_1019_MOESM6_ESM.pdf]

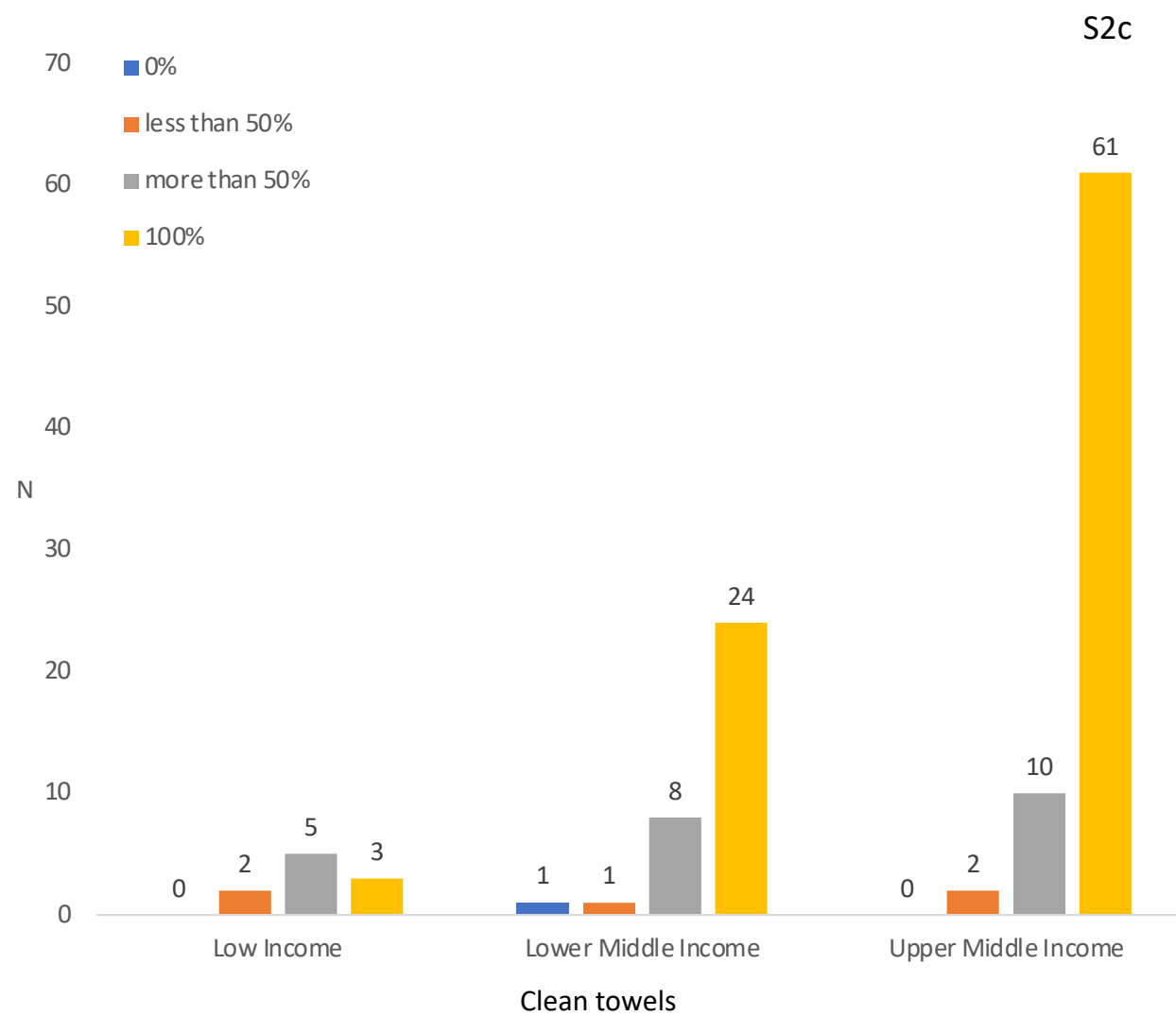

Supplement: Supplementary file 7 — Supplementary Figure 2c [file 41372_2021_1019_MOESM7_ESM.pdf]

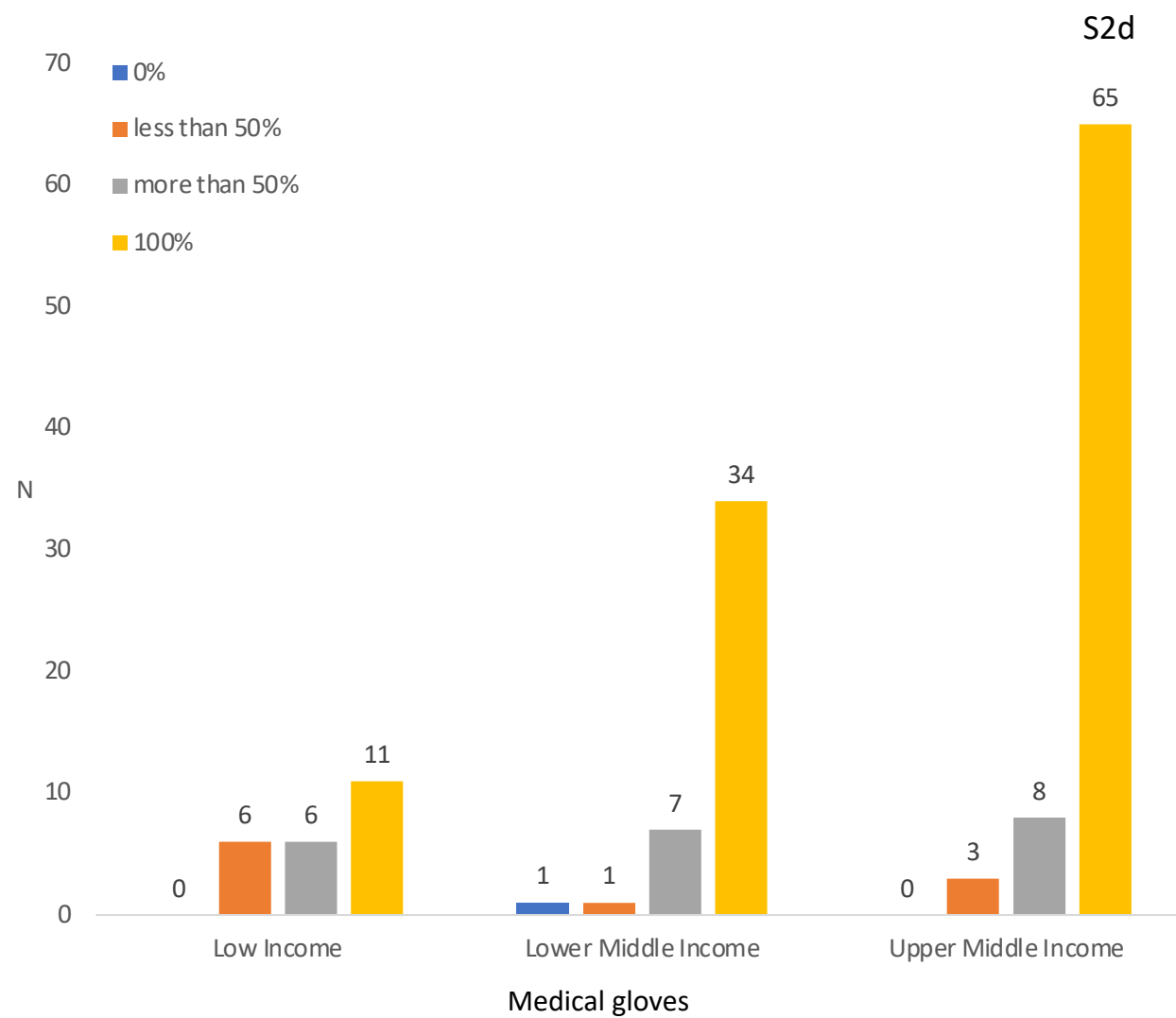

Supplement: Supplementary file 8 — Supplementary Figure 2d [file 41372_2021_1019_MOESM8_ESM.pdf]

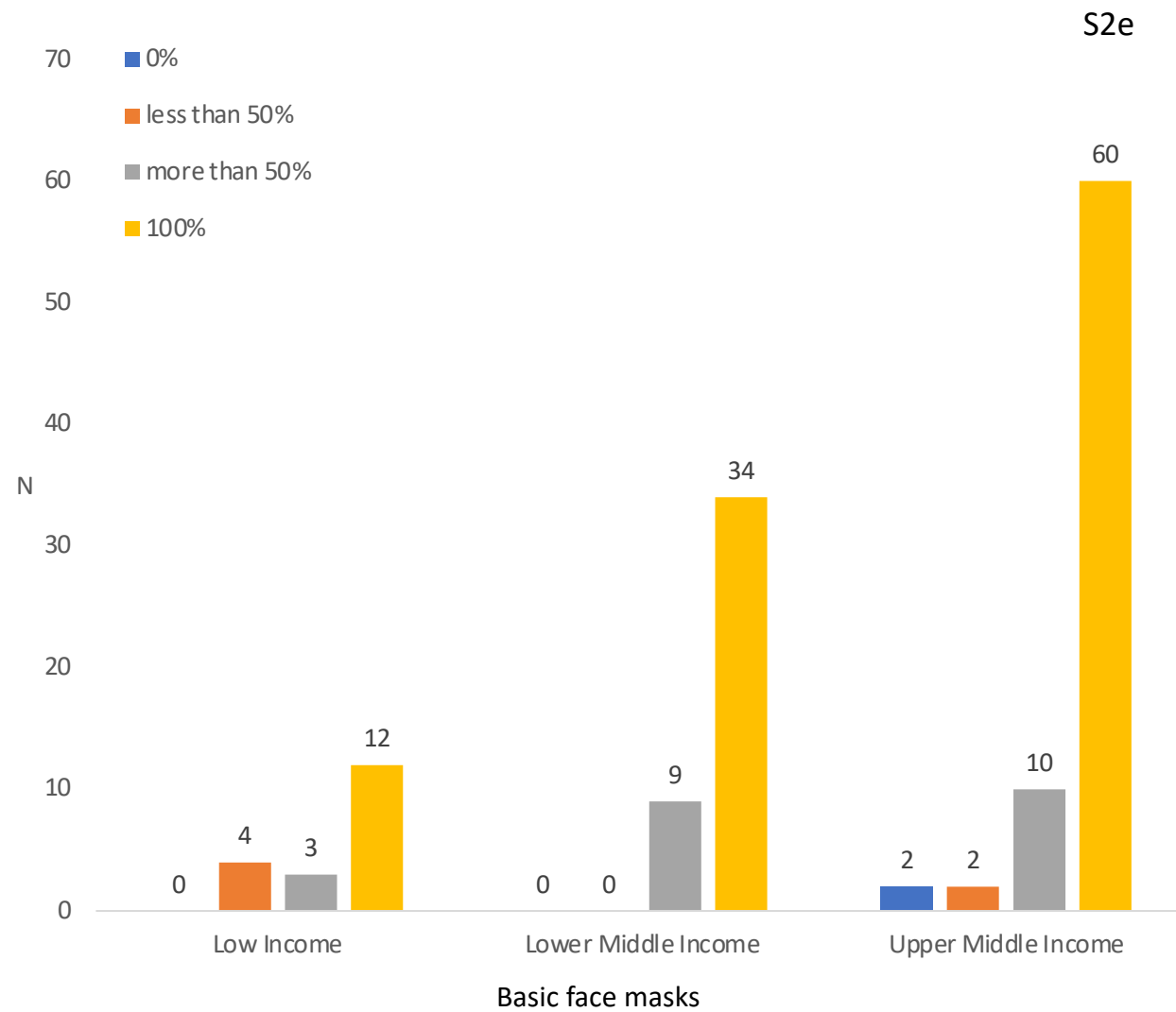

Supplement: Supplementary file 9 — Supplementary Figure 2e [file 41372_2021_1019_MOESM9_ESM.pdf]

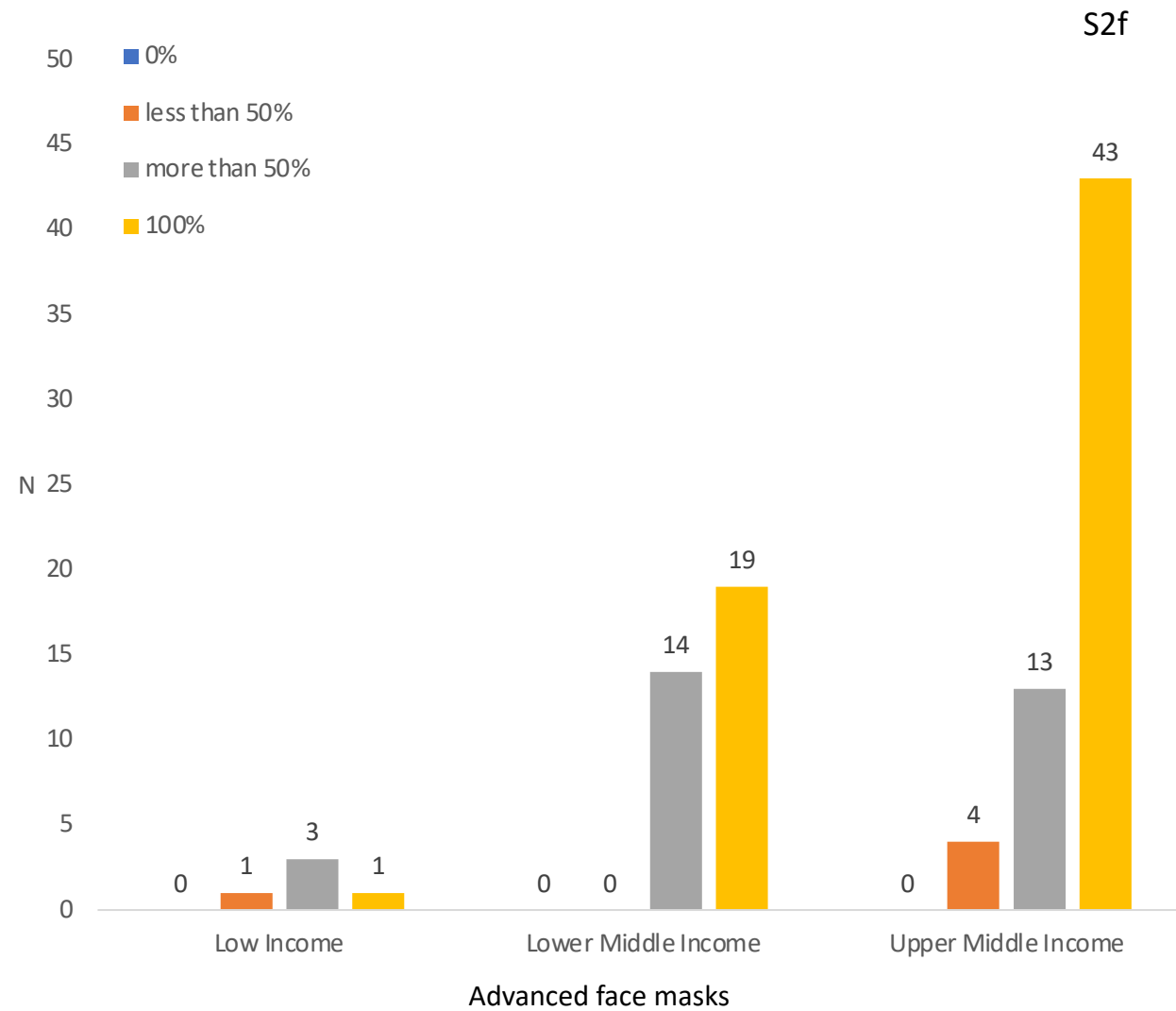

Supplement: Supplementary file 10 — Supplementary Figure 2f [file 41372_2021_1019_MOESM10_ESM.pdf]

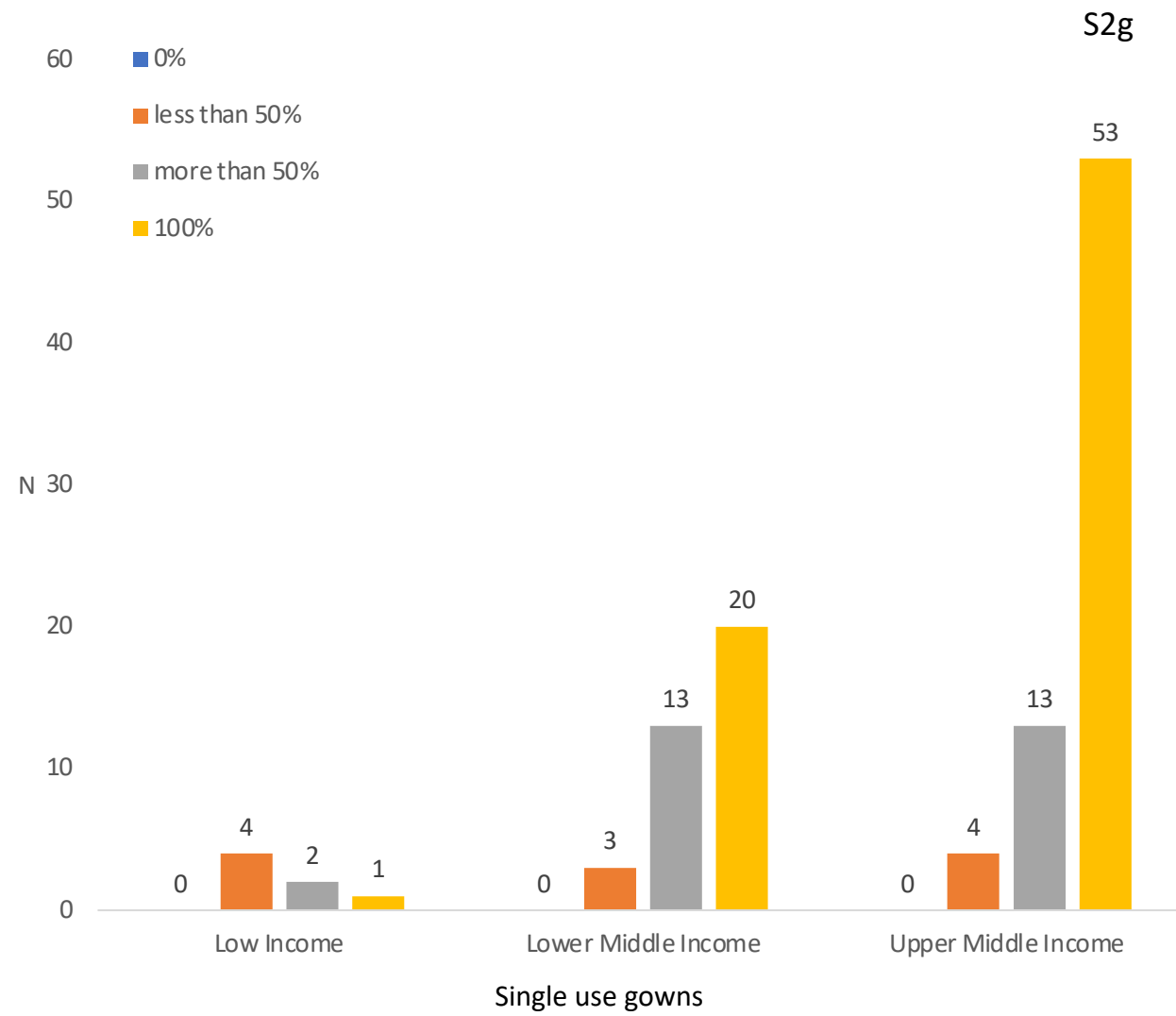

Supplement: Supplementary file 11 — Supplementary Figure 2g [file 41372_2021_1019_MOESM11_ESM.pdf]

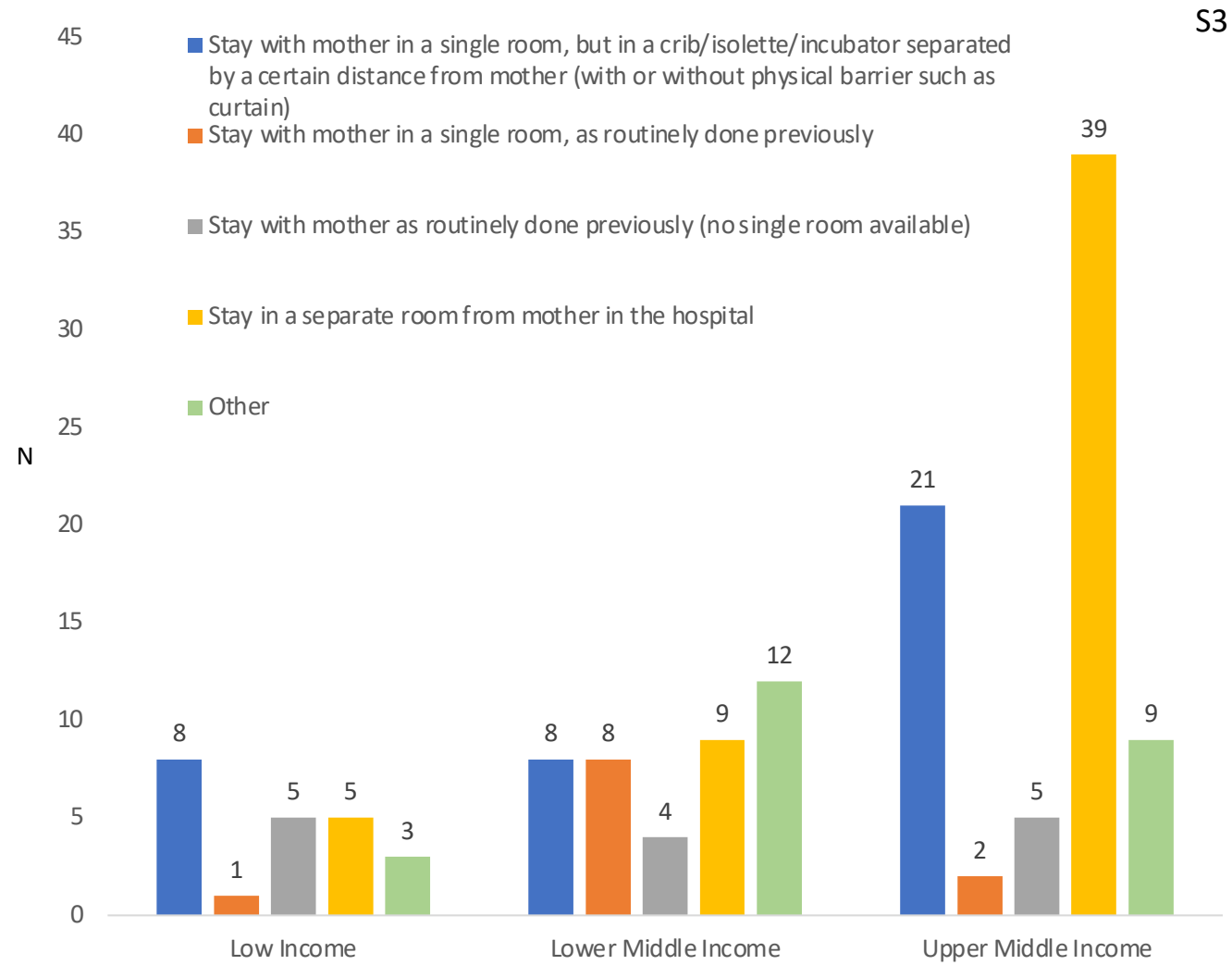

Where do you care for the asymptomatic infant born to a mother with COVID-19 initially after birth?

Supplement: Supplementary file 12 — Supplementary Figure 3 [file 41372_2021_1019_MOESM12_ESM.pdf]
