# Supplementary material for: COVID-19 preparedness—a survey among neonatal care providers in low- and middle-income countries
Source: J Perinatol. 2021 Apr 13;41(5):988–97. doi: 10.1038/s41372-021-01019-4 (PMC8042838; doi:10.1038/s41372-021-01019-4)
Supplement: Supplementary file 13 — Supplementary Figure 4 [file 41372_2021_1019_MOESM13_ESM.pdf]

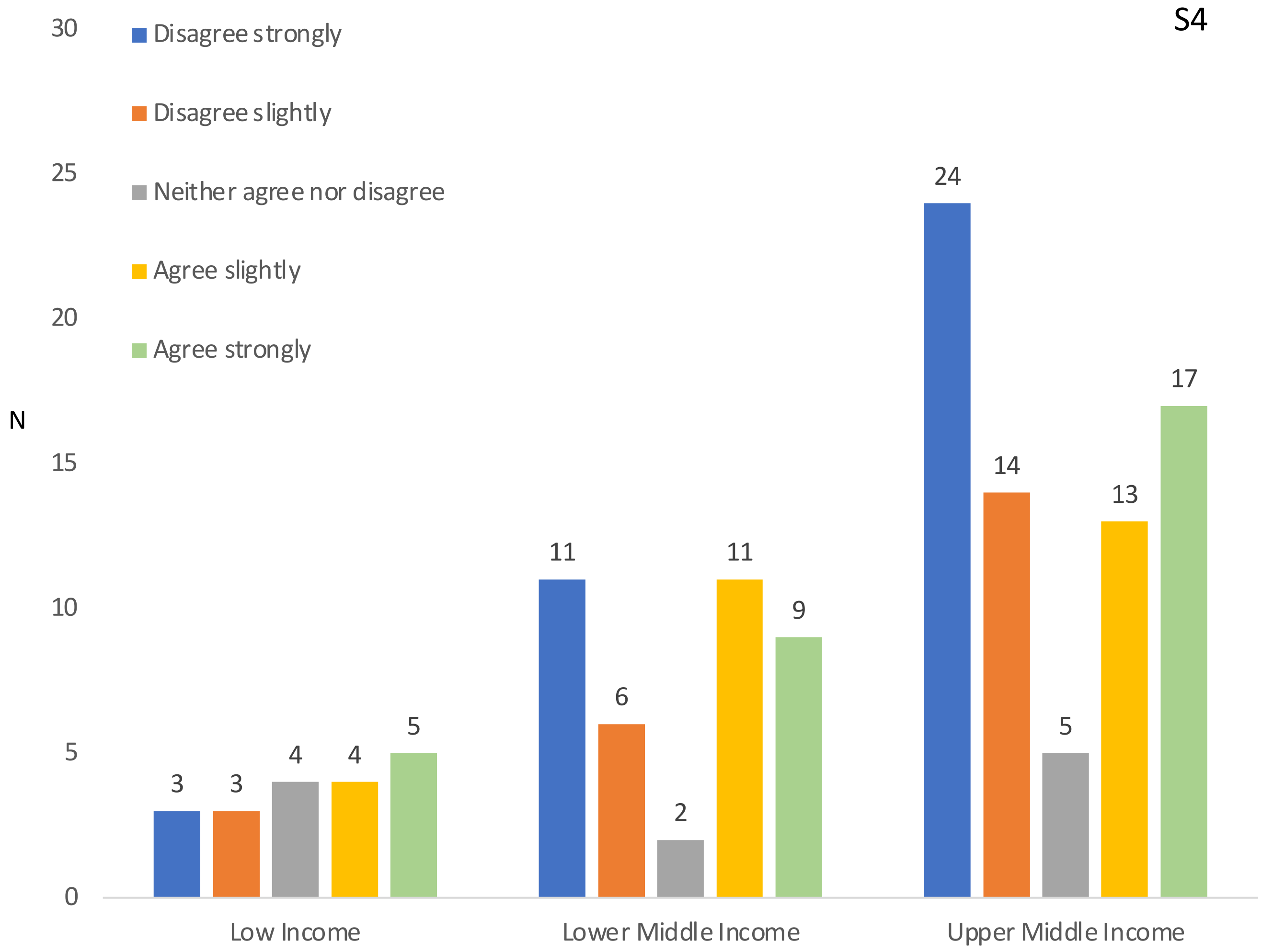

For the asymptomatic infant born to a mother with COVID-19: In our maternity/neonatal unit, we follow mother's/family's wishes regarding whether their infant will stay with mother after birth.
