# Supplementary material for: COVID-19 preparedness—a survey among neonatal care providers in low- and middle-income countries
Source: J Perinatol. 2021 Apr 13;41(5):988–97. doi: 10.1038/s41372-021-01019-4 (PMC8042838; doi:10.1038/s41372-021-01019-4)
Supplement: Supplementary file 15 — Supplementary Figure 5b [file 41372_2021_1019_MOESM15_ESM.pdf]

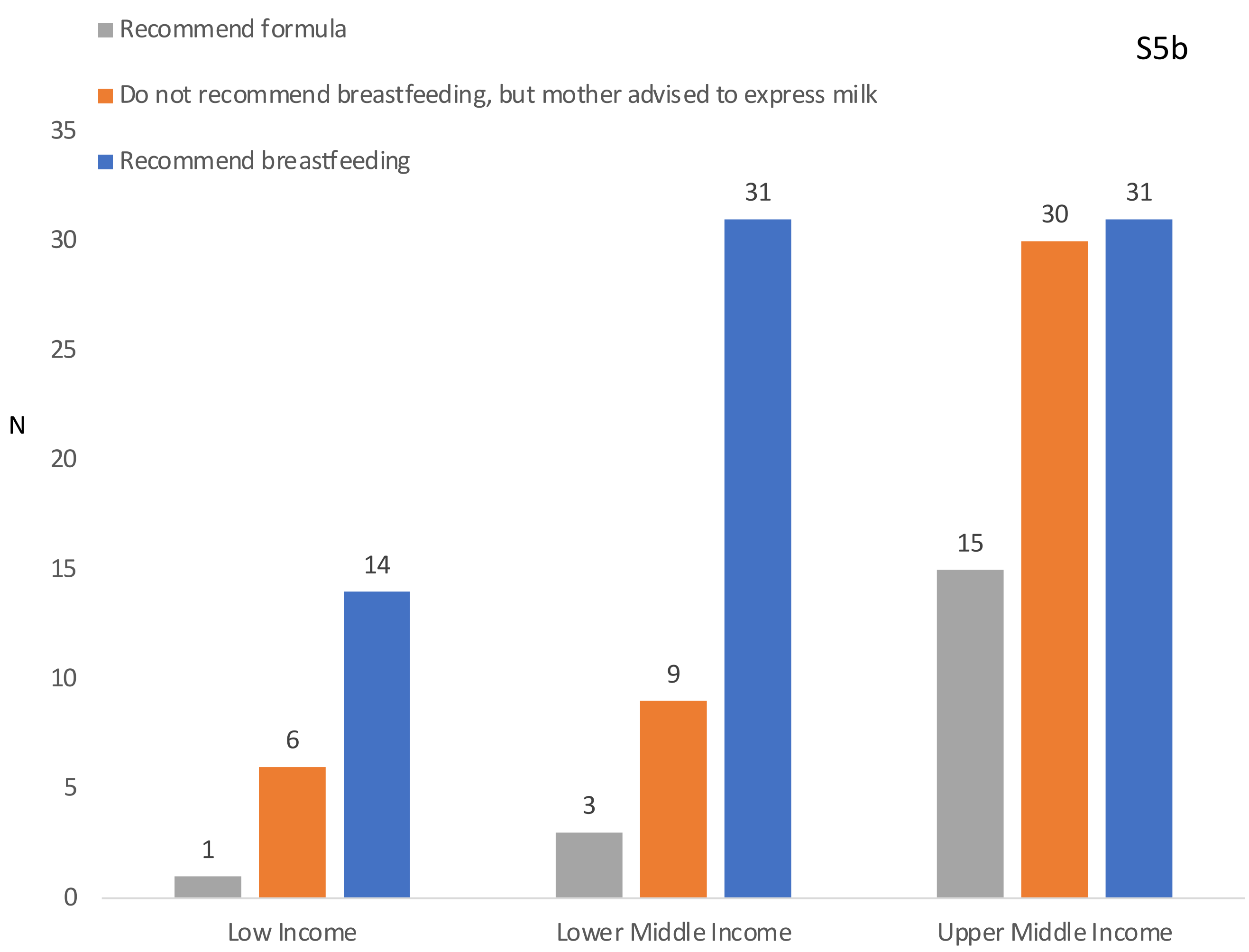

Feeding of asymptomatic infants born to SARS-CoV-2 positive or suspected positive mothers presented by income classification
