# Supplementary material for: COVID-19 preparedness—a survey among neonatal care providers in low- and middle-income countries
Source: J Perinatol. 2021 Apr 13;41(5):988–97. doi: 10.1038/s41372-021-01019-4 (PMC8042838; doi:10.1038/s41372-021-01019-4)
Supplement: Supplementary file 16 — Supplementary figure legends [file 41372_2021_1019_MOESM16_ESM.docx]

**iv) Supplementary Figure 1:** Classification of 145 responses by income level of countries in the five global regions

***Footnote to Supplementary Figure 1:***

**Low-income countries; LICs (n=11):** Burundi, Democratic Republic of Congo, Ethiopia, Malawi, Nepal, Rwanda, Somalia, Tajikistan, Tanzania, Uganda, Yemen

**Lower middle-income countries; lower-MICs (n= 22):** Bolivia, Cameroon, Egypt, Ghana, Honduras, India, Indonesia, Kenya, Kyrgyzstan, Lao PDR, Myanmar, Nicaragua, Nigeria, Pakistan, Senegal, Sudan, Tunisia, Ukraine, Uzbekistan, Vietnam, Zambia, Zimbabwe

**Upper middle-income countries; upper-MICs (n=25):** Albania, Algeria, Argentina, Azerbaijan, Belarus, Bosnia and Herzegovina, Brazil, Colombia, Dominican Republic, Ecuador, Guatemala, Iran, Iraq, Jordan, Lebanon, Libya, Malaysia, Mexico, North Macedonia, Paraguay, Peru, Russia, South Africa, Thailand, Turkey

**v) Supplementary Figures 2a – 2g:** Access to and availability of water, sanitation and personal protective equipment reported by respondents from 58 LMICs, classified by income category

- Running water
- Soap
- Clean towels
- Medical gloves
- Basic face masks
- Advanced face masks
- Single use gowns

**vi) Supplementary Figure 3:** Responses to survey question:

“Where do you care for the asymptomatic infant born to a mother with COVID-19 initially after birth?”

**vii) Supplementary Figure 4:** Responses to survey question:

“For the asymptomatic infant born to a mother with COVID-19: In our maternity/neonatal unit, we follow mother’s/family’s wishes regarding whether their infant will stay with mother after birth.”

**viii) Supplementary Figure 5:**

Feeding of asymptomatic infants born to SARS-CoV-2 positive or suspected positive mothers.
(presented by a) region and b) income classification)
